# The Role of Diet in Functional Dyspepsia

**DOI:** 10.3390/nu18183064

**Published:** 2026-09-19

**Authors:** Luisa Bertin, Cedric Van de Bruaene, Elena Formisano, Marcella Pesce, Stefania Piccirelli, Daniele Salvi, Karen Routhiaux, Andrea Pasta, Francesco Calabrese, Federico Caldart, Salvatore Crucillà, Giovanni Sarnelli, Elisa Marabotto, Javier Chahuan, Tom van Gils, Edoardo Vincenzo Savarino

**Affiliations:** 1 Department of Surgery, Oncology and Gastroenterology (DiSCOG), University of Padua, Via Giustiniani 2, 35128 Padua, Italy; luisa.bertin.1@phd.unipd.it; 2 Translational Research Center for Gastrointestinal Disorders (TARGID), Department of Chronic Diseases and Metabolism (CHROMETA), KU Leuven, Herestraat 49, 3000 Leuven, Belgium; 3 Nutritional Unit, Department of Internal Medicine, University of Genoa, Viale Benedetto XV 6, 16132 Genoa, Italy; 4 IRCCS Azienda Ospedaliera Metropolitana (AOM), Ospedale Policlinico San Martino, Largo Rosanna Benzi 10, 16132 Genoa, Italy; 5 Department of Clinical Medicine and Surgery, University of Naples Federico II, Via S. Pansini 5, 80131 Naples, Italy; 6 Digestive Endoscopy and Gastroenterology, Fondazione Poliambulanza Istituto Ospedaliero, Via Bissolati 57, 25124 Brescia, Italy; 7 Gastroenterology Unit, Department of Internal Medicine, University of Genoa, Viale Benedetto XV 6, 16132 Genoa, Italy; 8 Gastroenterology Unit, IRCCS Azienda Ospedaliera Metropolitana (AOM), Ospedale Policlinico San Martino, Largo Rosanna Benzi 10, 16132 Genoa, Italy; 9 Gastroenterology Unit, Department of Medicine, Pancreas Institute, University of Verona, Piazzale L.A. Scuro 10, 37134 Verona, Italy; 10 Departamento de Gastroenterología, Facultad de Medicina, Pontificia Universidad Católica de Chile, Santiago 8330077, Chile; 11 Department of Molecular and Clinical Medicine, Institute of Medicine, Sahlgrenska Academy, University of Gothenburg, 40530 Gothenburg, Sweden

**Keywords:** functional dyspepsia, postprandial distress syndrome, epigastric pain syndrome, diet, dietary patterns, low-FODMAP diet, Mediterranean diet, dietary fat, avoidant/restrictive food intake disorder, nutritional management

## Abstract

Functional dyspepsia (FD) is a disorder of gut–brain interaction affecting approximately 7% of the global population under Rome IV criteria, characterised by postprandial fullness, early satiation, epigastric pain, and/or epigastric burning in the absence of identifiable organic disease. Meal ingestion is the dominant symptom trigger in the majority of patients, yet the relationship between diet and FD is bidirectional: dietary exposures drive symptoms through established pathophysiological mechanisms, while FD itself can promote maladaptive eating behaviours and progressive dietary restriction with nutritional and psychological consequences. The pathophysiological substrate linking diet to symptom generation encompasses impaired gastric accommodation and emptying, visceral hypersensitivity to mechanical and chemical stimuli, low-grade duodenal mucosal immune activation and epithelial barrier impairment, alterations of the small intestinal microbiota, and gut–brain axis dysregulation. Dietary fat is the macronutrient most consistently implicated, acting through cholecystokinin-mediated chemosensory and mechanosensory pathways. Cross-sectional population data suggest that a high protein intake may also be associated with epigastric pain, although this association did not persist after multivariable adjustment, while the evidence linking total carbohydrate intake to symptoms remains sparse and inconsistent, so that no recommendation on total carbohydrate intake can currently be made. Cognitive factors, including learned food beliefs, expectancy, and nocebo effects, further modulate symptom perception independently of actual nutrient content. Among dietary patterns, Mediterranean-style eating offers the most coherent mechanistic rationale and the strongest observational support, while a diet low in fermentable oligosaccharides, disaccharides, monosaccharides, and polyols (FODMAP) shows selective benefit in patients with postprandial distress syndrome features or prominent bloating. Common triggers including spicy foods, coffee, and alcohol show heterogeneous evidence driven by individual sensitivity rather than dose-related effects. Nutritional concerns are substantial: clinically significant weight loss affects approximately 35% of tertiary-care patients, and nearly 40% screen positive for avoidant/restrictive food intake behaviour using validated screening tools. Dietary management should therefore be individualised, nutritionally adequate, and integrated within a broader biopsychosocial framework, with screening for disordered eating before any elimination strategy is initiated.

## 1. Introduction

Worldwide, many individuals experience bothersome postprandial fullness, early satiation, epigastric pain and/or epigastric burning that impair daily functioning. When these gastroduodenal symptoms cannot be explained by an identifiable organic disease and meet the recently published Rome V diagnostic criteria, the condition is classified as functional dyspepsia (FD) [1]. FD is a common disorder, with an estimated global pooled prevalence of 6.8% under Rome IV criteria, with variations between developed and developing countries [2], and it also occurs more frequently in women [3,4].

**Figure 1:**
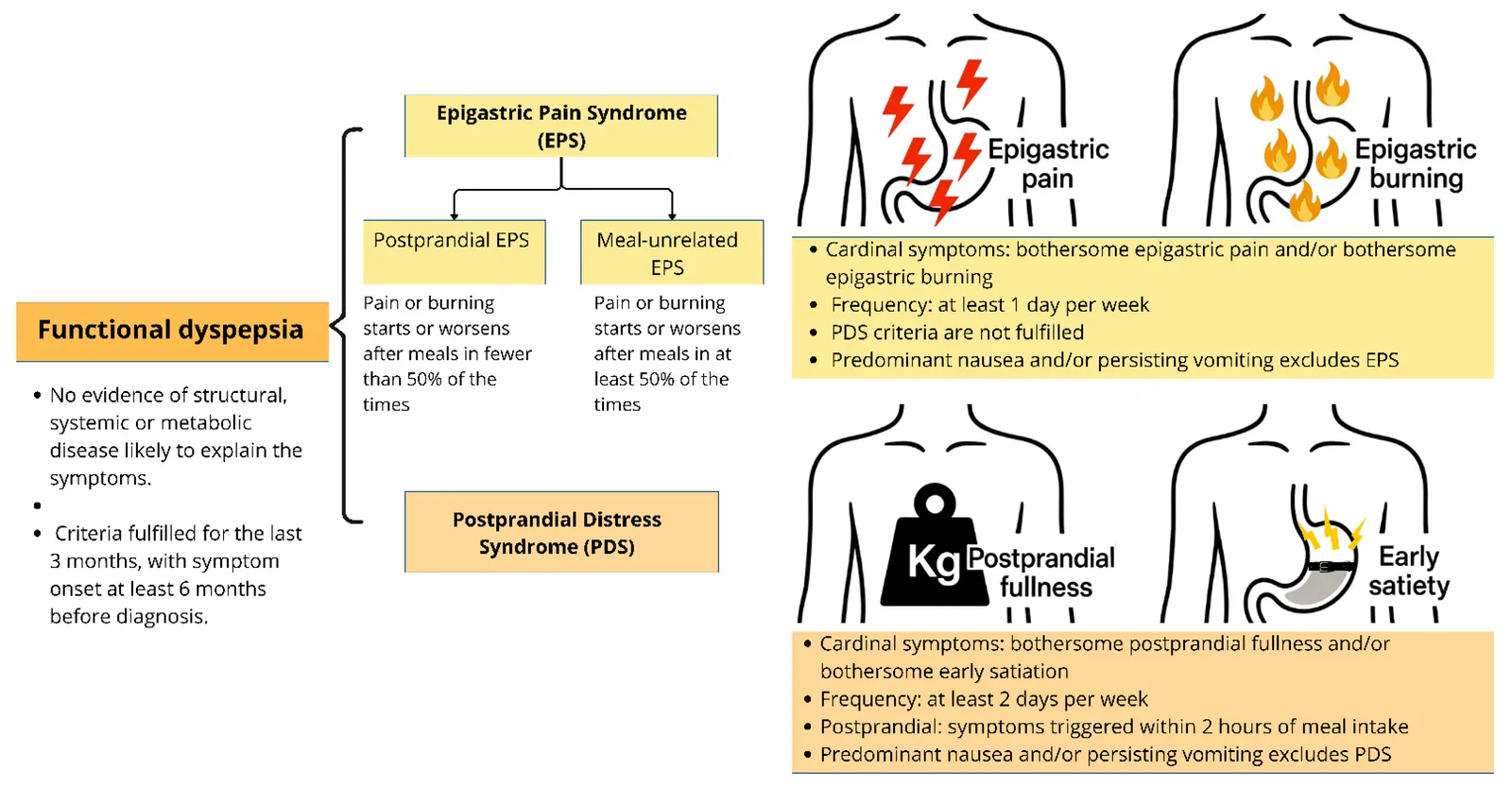
Classification of functional dyspepsia under the Rome V framework. Functional dyspepsia comprises epigastric pain syndrome, with the provisional postprandial and meal-unrelated forms characterised by epigastric pain or burning, and postprandial distress syndrome, characterised by postprandial fullness and early satiation. The diagnostic criteria that apply to each subtype are shown alongside the corresponding symptom categories. In the presence of the cardinal symptoms of postprandial distress syndrome, other bothersome symptoms elicited or worsened by food ingestion, namely postprandial epigastric pain or burning, nausea, upper abdominal bloating and excessive belching, are also regarded as part of postprandial distress syndrome. Abbreviations: EPS: epigastric pain syndrome; PDS: postprandial distress syndrome.

Based on predominant symptoms, FD is traditionally subdivided into postprandial distress syndrome (PDS), characterised by meal-related postprandial fullness and/or early satiation and epigastric pain syndrome (EPS), characterised by epigastric pain and/or burning that can be induced or occur independently from meal ingestion [1,5]. Rome V further subdivides EPS occurring without PDS into a postprandial and a meal-unrelated form, for which provisional criteria have been proposed according to whether the pain or burning starts or worsens after meals in at least half of the episodes. This symptom-based classification, together with the corresponding Rome V diagnostic criteria, is shown in Figure 1.

In the evaluation of dyspeptic patients, *Helicobacter pylori* infection should be ruled out non-invasively and eradicated if detected, since *Helicobacter pylori*-associated gastritis may mimic FD [1,6]; however, the benefit of eradication for symptoms resolution is modest [7]. If symptoms persist, FD should be considered [1]. In clinical practice, endoscopy is generally not mandatory in patients with suspected FD without alarm features or risk factors [1].

Although dietary advice are considered initially in treatment algorithms [1], the evidence supporting dietary therapy in FD remains limited, and patients are generally advised to consume small, frequent meals and avoid potential trigger foods such as spicy or fatty foods, coffee, carbonated beverages and alcohol [8]. However, the relationship between FD and diet is considerably more complex. FD itself may induce maladaptive changes in meal patterns and eating behaviours, potentially affecting nutrient intake and promoting restrictive dietary practices. Therefore, this review aims to provide a comprehensive overview of FD pathophysiology, eating behaviours and meal patterns, nutrient intake, evidence-based dietary interventions, commonly reported trigger foods and beverages and potential risks of dietary strategies.

### Literature Search, Evidence Framework and Scope

This article is a narrative review. A structured literature search was performed in PubMed/MEDLINE, Embase and the Cochrane Library from database inception to 31 May 2026, combining the terms functional dyspepsia, dyspepsia, postprandial distress syndrome and epigastric pain syndrome with diet, dietary pattern, meal pattern, meal frequency, macronutrient, fat, protein, gluten, wheat, carbohydrate, FODMAP, fibre, Mediterranean diet, DASH, ultra-processed food, food intolerance, trigger food, nutritional status and avoidant or restrictive food intake disorder. Original studies, systematic reviews, meta-analyses and guideline documents published in English and conducted in adults were eligible, and the reference lists of the retrieved articles and of recent guidelines were hand-searched for further sources. Selection was made by consensus among the authors, with priority given to controlled physiological experiments, randomised trials and large population-based cohorts. Because this is a narrative rather than a systematic review, no formal risk-of-bias assessment and no quantitative synthesis were performed, and selection bias cannot be excluded.

Two features of this literature should be kept in mind. First, it is geographically unbalanced. Second, the Rome V framework is used throughout as the reference definition of FD, since it is the criterion set now in force. Studies performed before its publication cannot be reclassified retrospectively, and the criteria actually applied in each cited study, whether Rome II, Rome III, Rome IV or a non-Rome definition of uninvestigated dyspepsia, are therefore stated in the text wherever this affects interpretation.

## 2. Food-Related Pathophysiological Mechanisms of Functional Dyspepsia

**Figure 2:**
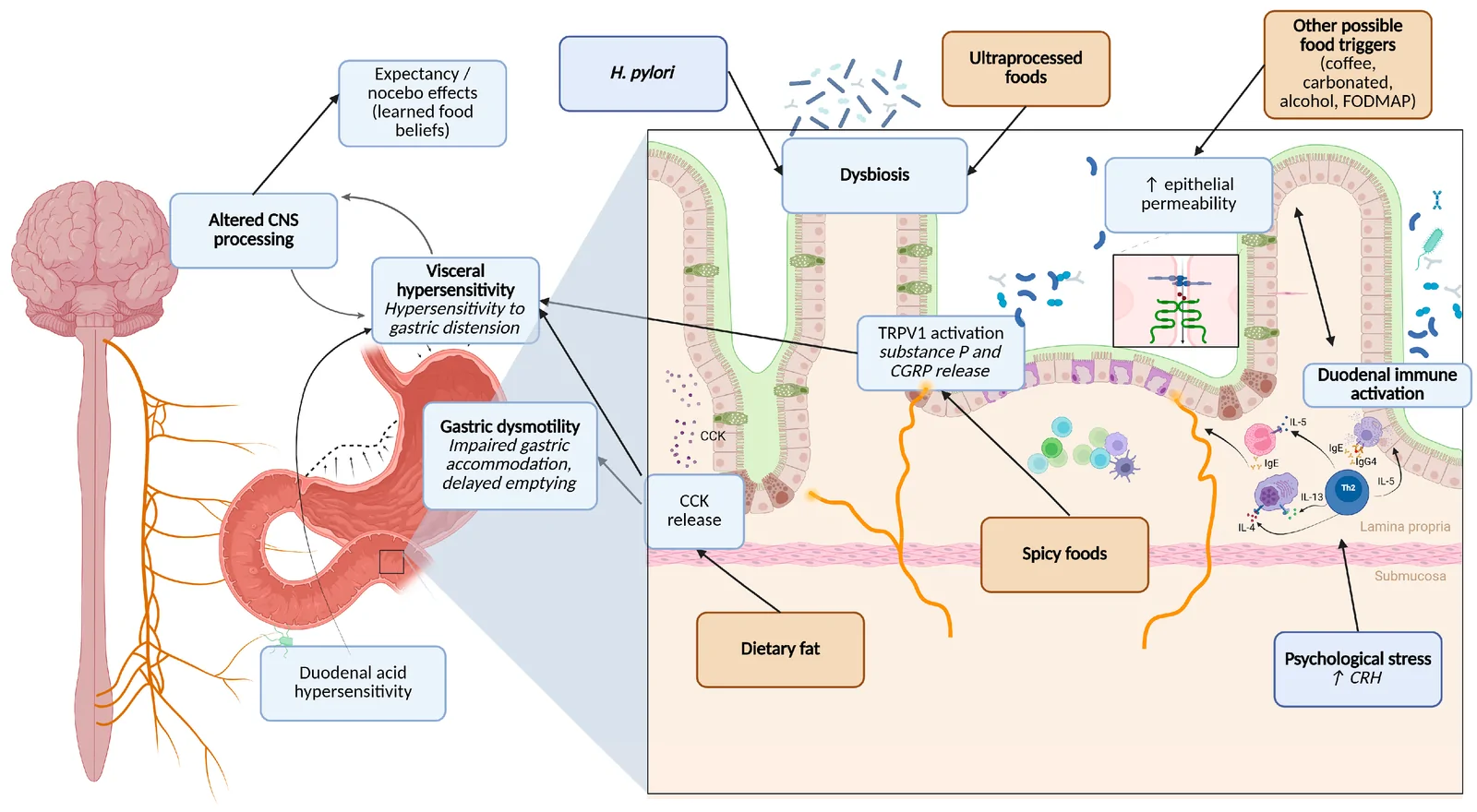
Food-related pathophysiological mechanisms of functional dyspepsia. Dietary triggers (dietary fat, spicy foods, fast foods, food antigens) and *H. pylori*-driven dysbiosis act at the duodenal mucosa to increase epithelial permeability and activate mucosal immune responses, driving visceral hypersensitivity and gastric dysmotility that are relayed through peripheral and vagal pathways to the central nervous system, where altered central processing and expectancy or nocebo effects (learned food beliefs) shape symptom perception. *H. pylori* is shown as a driver of dysbiosis in patients with persistent dyspeptic symptoms after successful eradication; symptomatic *H. pylori* infection responding to eradication is classified as *H. pylori*-associated dyspepsia and not FD. Created with BioRender.com. Arrows indicate the direction of the signalling pathways described in the text. Abbreviations: CCK: cholecystokinin; CGRP: calcitonin gene-related peptide; CNS: central nervous system; CRH: corticotropin-releasing hormone; IgE: immunoglobulin E; IL: interleukin; Th2: T helper 2; TRPV1: transient receptor potential vanilloid 1.

The pathogenesis of FD is multifactorial. Gastric sensorimotor dysfunction, visceral hypersensitivity, low-grade duodenal mucosal immune activation, epithelial barrier impairment, alterations of the small intestinal microbiota, and dysregulated gut–brain signalling each contribute in different combinations across patients [9,10,11,12,13,14]. This integrated framework probably applies to a substantial proportion of patients meeting symptom-based criteria for FD, with individual differences in the relative weight of each mechanism [14]. Meal ingestion is the dominant symptom trigger in the majority of patients, and this section is therefore organised around each mechanism with explicit consideration of its dietary engagement (Figure 2).

### 2.1. Gastrointestinal Motor and Sensory Dysfunction

Two long-recognised gastric motor abnormalities are impaired meal-induced fundic accommodation and abnormal gastric emptying. Impaired accommodation was originally documented in 40% of FD patients by gastric barostat and confirmed in 37% of a Rome III FD cohort [15,16], with no significant differences across PDS, EPS, and overlap subgroups. Delayed solid gastric emptying is present in 23–34% of large cohorts with inconsistent correlation with symptom profile [17,18,19]. In a single multicentre study of tertiary care patients with chronic upper gastrointestinal symptoms (observational evidence), patients with FD and patients with gastroparesis were reported to be indistinguishable in terms of symptoms, gastric emptying and pathological features, including loss of gastric interstitial cells of Cajal and of CD206-positive macrophages, which led the authors to propose that the two conditions form part of a common spectrum of gastric neuromuscular dysfunction [20]. The histological comparison, however, rested on full-thickness gastric biopsies from only nine patients with FD, nine with idiopathic gastroparesis and nine controls. This is therefore a proposed model derived from one observational dataset rather than an established conclusion, and it has not yet been replicated in an independent cohort.

Visceral hypersensitivity is the second major sensorimotor abnormality and is particularly relevant to the relationship between meals and symptoms. Hypersensitivity to gastric distension is present in approximately one-third of patients (34–37% across the largest barostat cohorts) [21,22], whereas chemosensitivity to luminal nutrients is more prevalent, occurring in up to 60–70% of patients and correlating with meal-related symptom severity [22,23]. The mechanistic role of dietary lipid has been particularly well characterised. Intraduodenal infusion of lipid, but not isocaloric glucose, increases gastric mechanosensitivity and provokes fullness, nausea, and epigastric discomfort even without concurrent gastric distension, demonstrating that chemosensitivity operates as an independent mechanism distinct from mechanosensory pathways [24,25]. Symptoms are attenuated by the cholecystokinin-A receptor antagonist dexloxiglumide, with a clear dose–response between intraduodenal lipid load, plasma cholecystokinin (CCK) concentrations, and symptom severity [25,26]. Because CCK rises comparably in FD patients and healthy controls during lipid challenge, the prevailing interpretation is that FD is characterised by hypersensitivity to physiologically normal CCK signalling rather than by excessive peptide release [25,26,27]. Both fasting and postprandial peptide YY release are diminished in FD, potentially further compromising satiety regulation [26,27]. These mechanistic infusion data are corroborated by isocaloric meal challenges: a high-fat (32 g fat, 500 kcal) yoghurt elicits significantly greater nausea and pain than an equicaloric high-carbohydrate yoghurt in FD patients [27]. Capsaicin, the principal pungent component of red pepper, activates the duodenal transient receptor potential vanilloid type-1 (TRPV1) channel on sensory afferents and triggers release of substance P and calcitonin gene-related peptide; approximately half of FD patients exhibit chemosensitivity to capsaicin challenge, and duodenal TRPV1 expression is upregulated [28,29]. Prolonged TRPV1 exposure paradoxically desensitises afferents through neuropeptide depletion, which provides the rationale for the observation that 2.5 g/day of red pepper powder for five weeks reduced epigastric pain, fullness, and nausea in a small placebo-controlled trial of 30 FD patients [30].

### 2.2. Immune Activation and Epithelial Barrier Dysfunction

The pathophysiological focus has progressively shifted to the duodenum, where structural and immune abnormalities appear central to symptom generation in a substantial subset of patients [10,13,31]. A systematic review and meta-analysis confirmed higher duodenal eosinophil counts in FD than in healthy controls, with stronger associations in post-infectious phenotypes; mast cell increases are less consistent [32]. Eosinophils and mast cells from FD mucosa show ultrastructural features of immune activation [33] and localise close to submucosal neurons, where fine nerve fibre sprouting correlates with the degree of eosinophil activation [34]. Reduced transepithelial electrical resistance and increased paracellular permeability are accompanied by reduced expression of zonula occludens-1, occludin, β-catenin, E-cadherin, and desmoglein-2, with barrier impairment correlating with eosinophil and mast cell density [35]. Increased epithelial gap density on confocal laser endomicroscopy (CLE, a probe-based technique that provides real-time microscopic imaging of the intestinal mucosa) and reduced in vivo mucosal impedance support barrier disruption [36,37]. Increased α4β7 + CCR9+ small bowel-homing T lymphocytes are associated with delayed gastric emptying and symptom intensity [38], and atopic and autoimmune backgrounds are more frequent in FD than in the general population [39]. Proton pump inhibitor treatment reduces duodenal eosinophils, mast cells, and hyperpermeability in parallel with symptom improvement, suggesting effects beyond gastric acid suppression [40]. In patients with IBS and negative food-specific IgE testing, CLE with topical duodenal application of food solutions (wheat, milk, soy, yeast and, in the later series, egg white) elicited acute epithelial breaks with fluorescein extravasation into the intervillous space in 76 of 108 patients (70%), most frequently after wheat; exclusion of the single food component that had reacted at CLE was followed by a mean reduction in symptom scores of approximately 70% at 3 months and 73% at 6 months, although these figures derive from uncontrolled follow-up rather than from a controlled comparison [41,42]. However, a sham-controlled randomised trial restricted to Rome IV FD subsequently found no clinical advantage of CLE-guided elimination over a sham diet, and CLE alterations did not correlate with objective barrier or immune markers, indicating that the clinical translation of this approach remains to be defined [43].

### 2.3. Microbiota Alterations

Alterations of the small intestinal microbiota provide a third axis linking diet to FD symptoms. Dysbiosis, defined as a structural or functional disruption of the mucosa-associated microbial community relative to that of healthy controls, has been characterised in FD as an increased duodenal bacterial load with relative expansion of *Streptococcus* and reduction of *Prevotella*, *Veillonella*, and *Actinomyces*, correlating positively with meal-related symptoms and inversely with quality of life [44]. Small intestinal bacterial overgrowth has been associated with FD in meta-analysis comparing FD to healthy controls; however, this association remains controversial due to the lack of standardised diagnostic criteria [45]. Diet directly shapes this compartment through fibre, fat, and fermentable Oligosaccharides, disaccharides, monosaccharides, and polyols (FODMAP) intake, which modify microbial composition and metabolite output. Post-infectious FD is an integrative clinical model: in a systematic review of 19 studies the mean prevalence of FD was approximately 9.6% after acute gastroenteritis, with approximately 2.5-fold increased odds of developing FD beyond six months [46]; *Salmonella, Escherichia coli, Campylobacter jejuni, Giardia lamblia,* and norovirus, most commonly acquired through contaminated food or water, and more recently SARS-CoV-2 have all been implicated [47,48]. Acute-onset FD, accounting for approximately 25% of tertiary care patients, shares duodenal eosinophil and CCR2+/CD68+ monocyte-derived macrophage infiltration with the post-infectious subset [49,50].

### 2.4. Gut–Brain Axis Dysregulation

Central and peripheral dysregulation along the gut–brain axis is the final and unifying mechanism of the disorder of gut–brain interaction framework within which FD is classified. Anxiety, depression, and extraintestinal somatic symptoms are highly prevalent, and longitudinal data demonstrate a bidirectional relationship in which psychological distress can precede gastrointestinal symptoms by several years, while persistent FD increases the subsequent risk of mood and anxiety disorders [51,52,53]. Corticotropin-releasing hormone increases small intestinal permeability in healthy volunteers through a mast cell-dependent pathway abrogated by disodium cromoglycate, linking psychosocial stress to the duodenal mucosal pathway described above [54]. Anxiety has been associated with duodenal eosinophilia in FD, and eosinophils themselves release CRH and substance P under stress [55,56]. Functional and structural neuroimaging consistently identifies abnormalities in interoceptive and affective processing regions including the insula, anterior cingulate cortex, prefrontal cortex, thalamus, and amygdala [57], and positron emission tomography has shown increased cerebral cannabinoid-1 receptor availability in FD, paralleling findings reported in eating disorders [58]. Crucially, expectancy and learned food beliefs, placebo and nocebo effects, are integral to how food triggers symptoms in this disorder. Functional MRI has shown that informing patients that a meal is high in fat modulates brain activity in interoceptive and reward-related regions in FD patients independently of actual macronutrient content [59]. In fact, in a randomised crossover study, low-fat yoghurt elicited levels of fullness and bloating similar to high-fat yoghurt when participants were incorrectly informed that the low-fat product was high-fat, confirming that symptom perception in FD is substantially modulated by learned food beliefs and anticipatory responses [60]. A comparable role for expectation has been shown for gluten: in a multicentre, randomised, double-blind, placebo-controlled trial in non-coeliac gluten sensitivity (NCGS), the combination of expectancy and actual gluten intake produced the largest effect on gastrointestinal symptoms, consistent with a dominant nocebo effect, although an additional effect of gluten could not be excluded [61]. These observations indicate that any dietary intervention in FD acts on the integrated mechanisms reviewed above through both physiological and cognitive pathways, and that recognising the nocebo component is essential for interpreting food–symptom associations and for designing rational dietary management strategies, as discussed in the subsequent sections.

### 2.5. From Luminal Nutrients to Cellular Events: How Dietary Stimuli Engage Gastroduodenal Neuromuscular Function

The mechanisms described above are usually reported at the level of organ physiology, but the steps that translate a luminal nutrient into altered gastric motor behaviour take place within an integrated cellular network. Gastric smooth muscle cells are electrically coupled to interstitial cells of Cajal (ICCs) and to platelet-derived growth factor receptor alpha-positive (PDGFRα+) cells, which together form the so-called SIP syncytium. Each interstitial cell type expresses a signature calcium-dependent conductance: ICCs express the calcium-activated chloride channel anoctamin-1, which generates inward current, whereas PDGFRα+ cells express small-conductance calcium-activated potassium channels, which generate an opposing outward current. The open probability of both conductances is governed by calcium release from the endoplasmic reticulum, and the resulting calcium transients arise spontaneously and stochastically from multiple sites within each cell. In pacemaker ICCs the transient inward currents and depolarisations they produce summate and activate voltage-dependent calcium influx, which in turn entrains calcium release into clusters and sustains activation of anoctamin-1 channels for the duration of the slow wave, so that the frequency and amplitude of contraction are set within the interstitial cell network rather than by the muscle itself; excitatory and inhibitory enteric motor neurons also act to a large extent through these interstitial cells rather than directly on smooth muscle [62]. Loss of ICCs and ultrastructural evidence of ICC injury, accompanied by an altered resident macrophage population, have been documented in full-thickness gastric body biopsies from 40 patients with diabetic or idiopathic gastroparesis and matched controls [63], and comparable pathological changes have been reported in patients with FD [20]. Full-thickness gastric tissue is rarely available in FD, so these observations are indirect and derive largely from the gastroparesis literature and from animal work. ICC dysfunction should therefore be regarded at present as a biologically plausible but unproven contributor to FD rather than as an established mechanism.

Against this background, the effect of dietary stimuli on gastric motor function is best understood as indirect and neurohormonally mediated rather than as a direct action of nutrients on smooth muscle or pacemaker cells. Meal-induced fundic relaxation is a vagovagal reflex whose efferent limb is nitrergic: in healthy volunteers, inhibition of nitric oxide synthase with N(G)-monomethyl-L-arginine reduced meal-induced relaxation from 274 mL to 82 mL at the higher dose and lowered the calorie load ingested at maximum satiation from 1058 to 892 kcal, identifying nitric oxide released from inhibitory enteric neurons as the final common mediator of accommodation (mechanistic evidence) [64]. Any dietary factor that weakens this reflex, or that increases the afferent traffic driving it, will lower the volume tolerated before fullness appears. The lipid is the best characterised such factor. Fatty acids released by intraduodenal lipolysis stimulate enteroendocrine I cells to secrete CCK, which acts on CCK1 receptors expressed on vagal afferent terminals. These are Gq-protein-coupled receptors that signal through phospholipase C, inositol trisphosphate and a rise in intracellular calcium, so that receptor occupancy increases afferent firing and, through vagovagal pathways, slows gastric emptying, reduces antral motility and increases pyloric tone [65]. Blockade of the CCK1 receptor with dexloxiglumide abolishes much of the symptomatic response to duodenal lipid in FD, and because plasma CCK rises comparably in patients and in controls the abnormality appears to lie in the gain of this afferent pathway rather than in the amount of peptide released [25,26]. Protein, and to a lesser extent carbohydrate, also releases CCK and other enteroendocrine mediators, which offers the most plausible explanation for the observation that postprandial fullness relates more closely to fat and protein intake than to carbohydrate intake [27].

Two further dietary inputs act on the sensory rather than on the motor side of the same network. Capsaicin activates TRPV1 on duodenal and gastric afferents, a non-selective cation channel whose opening produces calcium influx and release of substance P and calcitonin gene-related peptide; duodenal TRPV1 expression is increased in FD, and prolonged agonist exposure depletes these neuropeptides, which accounts for the paradoxical desensitisation seen with sustained rather than intermittent exposure [28,29]. Fermentable carbohydrates act mainly through osmotic water retention and bacterial fermentation, increasing luminal volume and gas and therefore the mechanical stimulus reaching an already hypersensitive wall [22,23]. In both cases the abnormality demonstrated in FD is amplified afferent signalling and altered central processing rather than a primary defect of the pacemaker apparatus, while mucosal changes such as eosinophil activation, sprouting of fine nerve fibres in the submucosal plexus and impaired barrier function provide a coherent route through which dietary and microbial antigens could sensitise this network [34]. Direct human evidence linking a specific dietary component to ICC function in FD is not currently available, and this represents a clear research priority.

## 3. Eating Patterns: Meal Timing and Meal-Skipping

How patients with FD organise their meals—how often, how regularly, how quickly, and in what volume they eat—is an independently modifiable dimension of dietary management distinct from food selection [1]. As mentioned earlier, the pathophysiological substrate linking these behaviours to symptoms is well established, with impaired gastric accommodation, delayed emptying, and heightened visceral sensitivity creating conditions in which the volume, timing, pace, and composition of meals directly influence intragastric pressure and sensory signalling [14]. The evidence underpinning recommendations on meal patterning has accumulated unevenly: a recent systematic review and meta-analysis of 11 observational studies (21,220 participants) provides the only synthesis of this literature to date, identifying higher meal frequency as the single dietary habit most reproducibly associated with reduced FD risk and confirming that the remainder of the available evidence derives from cross-sectional designs of variable quality [66]. The findings discussed below should therefore be interpreted as biologically plausible signals rather than as evidence of causation, since reverse causality, in which dyspeptic symptoms themselves drive maladaptive changes in meal patterning, cannot be excluded from cross-sectional data. A comprehensive synthesis of interventional and observational studies examining dietary habits in FD is provided in Supplementary Table S1 and Supplementary Table S2, respectively.

### 3.1. Meal Frequency and Regularity

Portion size and overall caloric content are key considerations in dietary management of FD. Patients commonly report that meals precipitate symptoms [14].

Meal frequency and the regularity of mealtimes have been examined most consistently. A large Iranian cross-sectional study of more than 4700 adults reported an inverse association between the number of daily meals and FD prevalence, with the lowest prevalence in those consuming three or more regular meals per day [67], and a Chinese study in 1304 rural participants confirmed an association of irregular mealtimes with both PDS and EPS [68]. The mechanistic interpretation is consistent with known mechanisms: in patients with impaired accommodation and delayed emptying, distributing the daily caloric load across more, smaller meals reduces the peak intragastric volume and the duration of postprandial gastric distension, both of which scale with the intensity of meal-related symptoms. Habitual omission of breakfast or lunch (defined as skipping on three or more days per week) has been positively associated with FD prevalence in two independent cross-sectional analyses [67,68], and a third cross-sectional analysis, in 8913 Japanese university students in whom FD was defined by Rome III criteria, found that skipping breakfast or lunch was positively associated with FD (adjusted odds ratios 1.60, 95% confidence interval 1.10 to 2.32, and 2.52, 95% confidence interval 1.04 to 5.18, respectively) and that meal frequency was inversely associated with its prevalence (adjusted odds ratio 2.72 for one meal per day and 1.69 for two meals per day, compared with three meals per day; p for trend 0.001) [69]. By contrast, a randomised trial in 53 patients meeting Rome IV criteria for PDS (interventional evidence) found that adding traditional dietary advice, namely small regular meals and a reduced intake of caffeine, alcohol, fizzy drinks and high-fat, processed or spicy foods, to reassurance and diagnostic explanation gave no additional benefit over four weeks (adequate relief of dyspeptic symptoms in 39% vs. 33%, *p* = 0.70) [70], a result the authors attributed in part to dietary modifications adopted spontaneously by participants before enrolment, which would attenuate the incremental effect of formal advice.

### 3.2. Eating Speed and Meal Size

The pace at which meals are consumed has likewise been examined in cross-sectional cohorts with concordant but heterogeneous findings. A study (*n* = 4763) reported that eating quickly, operationally defined as completing a main meal in under 10 min, was associated with a 42% higher odds of uninvestigated dyspepsia (dyspeptic symptoms not yet investigated by endoscopy to exclude organic disease) compared with eating slowly [67,71]; two smaller studies confirmed a positive direction of association with variable magnitude [72,73], the heterogeneity reflecting differences in how eating speed was measured and how outcomes were defined [67,69]. Nutrient drinking tests provide the mechanistic correspondence: patients with FD reach maximal satiety after ingesting a much smaller liquid meal than healthy controls (361 vs.1005 mL, that is 542 vs. 1508 kcal), indicating that rapid gastric loading exceeds the capacity for fundic relaxation and accelerates the onset of postprandial fullness and pain [74]. Slower eating, adequate mastication, and smaller bite sizes are therefore physiologically rational countermeasures, although their independent contribution has not been isolated in interventional studies, in which they are typically bundled with other components of standard dietary advice [75,76]. The role of intra-meal hydration is more nuanced: in the Iranian cohort, fluid intake during meals was not associated with uninvestigated dyspepsia in the general population, but a positive association did emerge in overweight individuals, suggesting that body composition may modulate the gastric response to combined solid–liquid loads [67]. Late-night eating and short meal-to-sleep intervals have been reported by some authors to associate with nausea and postprandial fullness [68], but not consistently replicated [67], and current evidence does not allow for a directional recommendation independent of overall meal patterning.

Two limitations should temper the translation of these observations into clinical practice. First, the dominance of cross-sectional designs precludes any inference about temporality: the question of whether disordered meal patterning precedes and contributes to the development of FD symptoms, or instead represents an adaptive response to existing symptoms, remains unresolved, and well-designed prospective and interventional studies specifically targeting meal patterning as an independent variable are needed. Second, contemporary dietary trials have rarely stratified outcomes by FD subtype, and a recent pooled analysis across four FD datasets demonstrated that approximately 40% of patients with epigastric pain syndrome report postprandial epigastric pain in the absence of PDS features, the pattern for which Rome V has since introduced the provisional category of postprandial EPS, challenging the implicit assumption that meal pattern interventions are clinically relevant only to the PDS phenotype and arguing for subtype-stratified dietary trials going forward [5]. Despite these caveats, structured meal patterning, regular timing, three balanced meals per day, avoidance of skipping breakfast or lunch, moderate portion sizes, and an unhurried eating pace, remains a low-risk, physiologically coherent component of FD management that is well aligned with current mechanistic understanding and should be integrated into the broader dietary and psychosocial care.

## 4. Macronutrients

### 4.1. Dietary Fat

Dietary fat is the macronutrient most consistently implicated in FD symptom generation, and its effects operate through both sensory and hormonal mechanisms [27,77,78,79]. Isocaloric duodenal lipid infusion, but not glucose infusion, provoked epigastric fullness and discomfort during gastric distension in FD patients, whereas healthy controls remained asymptomatic under both conditions [24].

The role of cholecystokinin (CCK) has been established through complementary work. Lipid-induced symptoms during gastric distension were significantly attenuated by the CCK-A receptor antagonist dexloxiglumide, confirming CCK-A receptor involvement [25]. A dose–response relationship was further demonstrated that higher lipid concentrations produce greater plasma CCK concentrations and more severe symptoms of fullness, epigastric discomfort, and nausea. The association between plasma CCK levels and symptom severity during lipid infusion was subsequently corroborated [26]. Notably, CCK concentrations rose similarly in both patients and healthy controls, indicating that hypersensitivity to physiologically normal CCK signalling underpins fat-induced symptom generation in FD. Additionally, altered peptide YY (PYY) secretion, reduced in FD patients relative to controls in response to fat, may contribute further to impaired postprandial satiety regulation. Importantly, both studies documented symptom exacerbations during lipid infusion even in the absence of concurrent gastric distension, demonstrating that chemosensitivity constitutes an independent mechanism distinct from mechanosensory signalling.

Although mechanistic infusion studies have inherent limitations, their findings are nevertheless corroborated by dietary intervention studies. A high-fat soup elicited greater epigastric pain and nausea than a low-fat preparation in FD patients; a high-fat yoghurt increased fullness, bloating, and nausea compared with a low-fat version [22,79]. A controlled meal study demonstrated significantly greater FD symptoms, particularly nausea and pain, with a high-fat isocaloric meal than with an equicaloric high-carbohydrate meal, confirming that it is fat composition rather than caloric content that drives symptom generation [27]. Meal palatability may exert additional modulatory effects [80]. It should be emphasised, however, that this evidence comes from duodenal lipid infusion experiments, single-meal comparisons and cross-sectional surveys, and that no randomised controlled trial has evaluated sustained fat restriction as a treatment for FD. Moderate fat reduction is therefore best regarded as a mechanistically plausible and low-risk measure that can be trialled on an individual basis and reassessed, rather than as an established treatment, and its long-term efficacy and nutritional safety in this population remain to be demonstrated.

### 4.2. Proteins, Including Gluten

Evidence on the contribution of total protein intake to FD symptoms has accumulated only recently and warrants explicit consideration. In the SEPAHAN study, a population-based cross-sectional survey of 4763 Iranian adults in whom uninvestigated dyspepsia was defined by a modified Rome III questionnaire (observational evidence), the highest category of protein intake (>20% of energy) was associated with a higher unadjusted prevalence of epigastric pain than the lowest category (≤15% of energy; 50% vs. 39.7%, *p* = 0.02), and this was the only macronutrient–symptom association to reach unadjusted statistical significance for epigastric pain specifically [81]. The association was not retained after multivariable adjustment (odds ratio 1.48, 95% confidence interval 0.84 to 2.58), and the same analysis found no significant association between any macronutrient and the prevalence of uninvestigated dyspepsia overall. However, both the unadjusted trend and the calculated symptom-predictive thresholds are consistent with an earlier prospective observational study in 20 patients with FD in which postprandial fullness was related directly to both fat and protein intake and inversely to carbohydrate intake [82].

Beyond total protein, wheat-derived proteins constitute a distinct dietary trigger in a clinically relevant subset of patients with FD. In an Italian prospective multicentre survey of 486 individuals with suspected NCGS, the clinical picture was characterised by a combination of gastrointestinal manifestations, including abdominal pain, bloating, nausea, epigastric pain, and gastro-oesophageal reflux, and systemic features such as tiredness, headache, and foggy mind, with onset typically within a few hours to one day after gluten ingestion; concurrent IBS and food intolerance were reported by 47% and 35% of patients respectively, while IgG anti-gliadin antibodies were detected in 25% [83]. In an Australian population-based study of 3542 adults (observational evidence), self-reported wheat sensitivity was present in 14.9% and was independently associated with FD (odds ratio 1.48, 95% confidence interval 1.13 to 1.94) and with IBS (odds ratio 3.55, 95% confidence interval 2.71 to 4.65) [84]. A systematic review with meta-analysis of five randomised trials found that gluten challenge significantly increased the severity of bloating (weighted mean difference 0.67, 95% confidence interval 0.37 to 0.97), early satiety (0.91, 95% confidence interval 0.58 to 1.23) and epigastric pain (0.46, 95% confidence interval 0.17 to 0.75), whereas nausea was not significantly affected. Heterogeneity was substantial for bloating and epigastric pain and the certainty of the evidence was graded as very low [85]. A pilot double-blind randomised placebo-controlled crossover trial (interventional evidence) that enrolled eleven patients with Rome III FD, of whom nine completed the elimination phase, combined gluten-free and low-FODMAP elimination and produced a non-significant overall reduction in symptoms (Nepean Dyspepsia Index score 71.2 vs. 47.1, *p* = 0.087), and no specific food trigger could be reliably demonstrated during the rechallenge phase, illustrating the methodological challenge of small underpowered trials in this area [86]. Current evidence does not support a gluten-free diet for FD as a whole. Available data therefore support a trial of wheat or gluten restriction only in selected patients and only after exclusion of coeliac disease and wheat allergy, with the caveat that expectancy effects may contribute substantially to the perceived clinical response, as demonstrated earlier in the multicentre randomised double-blind trial in NCGS [61].

### 4.3. Carbohydrates, Including FODMAPs

Evidence linking total carbohydrate intake to FD symptoms is sparse and inconsistent. Within dietary carbohydrates, the components most relevant to FD symptoms are fermentable oligosaccharides, disaccharides, monosaccharides and polyols (FODMAP), which are considered in detail in the section on exclusion diets. One study reported that women with functional dyspepsia had lower intakes of energy, carbohydrate, fat and vitamin C than controls, with no significant difference in men and no difference in protein intake in either sex [87], but the proportion of energy derived from carbohydrate was the same in patients and controls (44.7% vs. 45.5%), and the authors report that nutrient densities expressed per 10 MJ per day are very similar in female patients and controls, so these data reflect a lower overall energy intake rather than a specific association between total carbohydrate intake and functional dyspeptic symptoms. A case–control study found no significant difference in absolute daily carbohydrate intake between FD patients and healthy controls [88], although patients in that study derived a higher proportion of their energy from carbohydrate than controls (56% vs. 50%) and a lower proportion from fat (28% vs. 34%), and a third study found no relationship between high carbohydrate meal content and symptom induction [27]. In the SEPAHAN survey discussed above, participants with uninvestigated dyspepsia had a marginally lower carbohydrate intake than those without (48.2% vs. 49.2% of energy, *p* = 0.01), but an intake above 55% of energy was not significantly associated with dyspepsia either before or after multivariable adjustment (adjusted odds ratio 0.86, 95% confidence interval 0.64 to 1.16) [81]. No specific recommendation regarding total carbohydrate intake in FD can therefore be made.

## 5. Dietary Patterns and Specific Diets

Dietary counselling is a cornerstone of FD management, aiming to limit symptom triggers while maintaining nutritional adequacy [12,38,75,89]. Although patients commonly adopt food avoidance strategies and dietary modification may plausibly influence gastric accommodation and postprandial hypersensitivity, the supporting evidence remains limited. Both the British Society of Gastroenterology and the UEG/ESNM consensus underscore uncertainty regarding non-pharmacological treatments and recommend dietary interventions only when individualised and pursued cautiously [12,89]. Evaluating overall dietary patterns, rather than isolated macronutrients or individual foods, may therefore offer a more clinically useful framework than rigid allowed/forbidden food lists.

### 5.1. Mediterranean Diet

The Mediterranean dietary pattern (MD) offers a low-risk, metabolically favourable dietary framework supported by robust cardiometabolic evidence [90]. Centred on minimally processed plant foods, extra-virgin olive oil, nuts, and fish, and characterised by reduced intake of ultra-processed products (UPFs), the MD has been associated with lower circulating inflammatory biomarkers, gut microbiota profiles enriched in short-chain fatty acid (SCFA)-producing taxa (SCFAs are a key energy source for colonocytes, support epithelial barrier integrity, and exert anti-inflammatory effects), and more favourable indices of intestinal barrier function [91,92]. These associations are mechanistically coherent in the context of FD: combining lower saturated fat and UPF exposure with higher intakes of fibre, polyphenols, and monounsaturated and omega-3 fatty acids, the MD may attenuate low-grade duodenal inflammation, support epithelial barrier integrity, and promote beneficial microbial activity [93].

Among dietary patterns, MD adherence shows the most consistent observational evidence of benefit in FD. In the GRADE-rated systematic review by Ashoorion and colleagues, in which the estimate for the MD was contributed by a single low-risk-of-bias observational survey of 1134 participants (observational evidence), high vs. low MD adherence was associated with a reduced likelihood of FD (odds ratio 0.41, 95% confidence interval 0.25–0.68), corresponding to an absolute risk reduction of approximately 151 fewer FD cases per 1000 individuals (95% confidence interval 74–203 fewer), classified as moderate-quality evidence [94]. The MD’s main practical strengths in FD are its nutritional adequacy, safety, and flexibility: meal volume, fibre type, fat load, and fermentable carbohydrate exposure can be individualised to symptom tolerance, and it can be used as a background framework of dietary quality while patient-specific triggers are systematically identified [12,75,89]. No randomised controlled trial has, however, evaluated the MD as a treatment for FD, and the FD-specific estimate reported above derives from a single observational survey, in which reverse causation cannot be excluded because patients whose symptoms are triggered by meals may themselves move away from a Mediterranean pattern. The MD is therefore proposed here on the grounds of nutritional adequacy, safety and tolerability, and not as a dietary pattern of demonstrated efficacy in FD.

### 5.2. Low-FODMAP Diet

Among structured dietary interventions applied in disorders of gut–brain interaction, the low-FODMAP diet has accumulated the most direct trial data relevant to FD, particularly in patients with PDS features, prominent bloating, or FD–IBS overlap, though current guidelines still consider the evidence insufficient to recommend a specific diet for FD [95,96,97,98]. The low-FODMAP approach reduces short-chain carbohydrates that are poorly absorbed in the small intestine and subject to rapid bacterial fermentation in the colon, generating gas and osmotic luminal distension [96]. Whether FODMAP restriction also acts on the duodenal mucosa is less clear: in a 6-week low-FODMAP intervention in 36 patients with FD and predominantly PDS symptoms (interventional evidence), 73% reached a clinically meaningful improvement in the Leuven Postprandial Distress Scale, but duodenal transepithelial electrical resistance and macromolecular flux were unchanged, although the change in resistance correlated with the change in symptom score [99]. A cross-sectional study found that 55% of dyspepsia patients self-identified FODMAP as symptom triggers [100], and a systematic review identified that foods commonly reported as FD triggers, including wheat and grain products, soft drinks, fruit juice, and milk, are also high in FODMAP [101].

The clinical trial evidence, though limited, supports selective rather than universal benefit. A single-blind randomised trial that screened 184 patients and randomised 105 with Rome IV FD (interventional evidence) found that both a low-FODMAP diet and traditional dietary advice improved symptoms and quality of life, with no significant difference in overall response, although patients with postprandial distress and bloating derived greater benefit from the low-FODMAP approach [102]. A subsequent randomised trial confined to PDS found the low-FODMAP diet superior to traditional dietary advice over six weeks: a clinically meaningful fall in the PDS subscale of the Leuven Postprandial Distress Scale was reached by 61.3% vs. 37.9% at the 0.5-point threshold (*p* = 0.04) and by 61.3% vs. 20.7% at the 0.7-point threshold (*p* < 0.001), and adequate relief of dyspepsia was reported by 71.0% vs. 34.5% (*p* = 0.005) [103]. An observational study in a predominantly FD–IBS overlap cohort similarly found greater reductions in epigastric and overall gastrointestinal symptom scores with the low-FODMAP diet compared to standard advice [104]. An earlier study in 1372 patients with disorders of gut–brain interaction, including 606 with FD, found that more than 80% of those testing positive for fructose or lactose intolerance achieved substantial symptom relief following restriction of saccharides and polyols over 4 weeks [105], although this uncontrolled outcome was reported for the whole cohort of disorders of gut–brain interaction rather than separately for the 606 patients with FD. Systematic reintroduction of individual FODMAPs suggests that not all subclasses contribute equally: in a blinded randomised reintroduction study in patients with IBS, fructans (56%) and mannitol (54%) were the most prevalent triggers of symptom recurrence, followed by galacto-oligosaccharides (35%), lactose (28%), fructose (27%) and sorbitol (23%), with glucose (26%) serving as the control; symptom-severity scores rose significantly only after fructans and mannitol, and the overall pattern of recurrence was highly individualised, averaging 2.5 triggers per patient [106]. A blinded reintroduction phase carried out in patients with FD rather than IBS gave an equally individualised but different pattern: at least one FODMAP provoked symptom recurrence in 46% of patients, mannitol being the most frequent single trigger (23%), followed by fructans, galacto-oligosaccharides, fructose and sorbitol (15% each) and lactose (12%), while 27% reacted to the glucose control [99].

Given the overall quality of evidence remains low, the low-FODMAP diet should be considered a structured, time-limited therapeutic trial in selected patients, specifically those with PDS features, FD/IBS overlap, or bloating-predominant symptoms, followed by systematic reintroduction to identify the least restrictive long-term pattern compatible with symptom control. This approach minimises the nutritional risks of prolonged global FODMAP restriction and limits unfavourable effects on microbiota diversity. The pilot double-blind randomised crossover rechallenge trial already discussed in Section 4.2, which enrolled eleven patients of whom nine completed the elimination phase, found only modest, non-significant symptom improvement, and only four participants qualified for rechallenge, of whom three completed it, so that no specific trigger could be identified [86], illustrating the methodological challenges inherent in this research area.

### 5.3. Gluten-Free/Wheat-Restricted

In patients with refractory FD, after exclusion of coeliac disease and wheat allergy, 35% of patients improved during a 6-week gluten-free diet; of these responders, 18.5% experienced symptom recurrence during blinded gluten challenge, representing 6.4% of the overall refractory FD cohort [107]. A more recent multicentre trial found that 52.3% of refractory IBS/FD patients responded to a 6-week gluten-free diet, though only 20.1% experienced significant symptom worsening during blinded gluten challenge, meeting criteria for NCGS [108]. Independent predictors of confirmed wheat sensitivity included female sex, FD–IBS overlap, headache, fatigue, and anxiety, with IgG antigliadin antibodies. Collectively, these findings indicate that clinical benefit from gluten restriction is likely confined to a subset of patients, and that it remains difficult to attribute improvement to gluten specifically, given that gluten restriction simultaneously reduces exposure to wheat, and subsequently fructans and other bioactive wheat components [109,110].

### 5.4. Non-Established Dietary Interventions for FD

#### 5.4.1. CLE-Guided Elimination

A randomised double-blind crossover sham-controlled trial of confocal laser endomicroscopy-guided dietary elimination in 17 Rome IV FD patients found no clinical advantage over a sham diet (response rates 4/14 vs. 2/14, *p* = 0.41), and confocal alterations did not correlate with objective barrier or immune markers, indicating that this approach is not currently ready for clinical application [43].

#### 5.4.2. Plant-Based Diets

Vegetarian and vegan dietary patterns are increasingly adopted and can be nutritionally adequate, but evidence supporting them as FD-specific therapy is currently insufficient [111]. A recent systematic review judged the effect of vegetarian and vegan diets on PDS to be uncertain, owing to the very low quality of evidence and the limited number of available studies [94]. From a mechanistic perspective, plant-based diets may plausibly influence FD through anti-inflammatory effects and favourable microbiota-related pathways; however, they simultaneously increase fermentable carbohydrate and fibre intake, which may aggravate bloating and postprandial symptoms in patients with distension-sensitive phenotypes [112,113].

The clinical relevance of fibre in FD depends not only on quantity but on type and food structure. Higher fruit and vegetable intake has been associated with lower odds of dyspeptic symptoms [114], a finding that is notable given that some fruits are themselves high in FODMAP and suggests that overall dietary quality, rather than individual high-FODMAP foods, drives symptoms in many patients. However, an excessive fibre intake, or specific poorly tolerated fibre types, may worsen fullness, bloating, and discomfort, particularly in patients with delayed gastric emptying [112,113]. Viscous soluble fibres, such as oats, psyllium, and pectins, may further slow gastric emptying and are therefore less well tolerated when consumed in large amounts. Because there is no rationale for recommending a vegetarian or vegan diet specifically to manage FD, this discussion concerns patients who already follow a plant-based diet and subsequently develop FD symptoms. The key clinical question is therefore not whether plant-based diets could treat FD per se, but how to implement them, in patients who already follow a vegetarian or vegan diet, in a way that is nutritionally adequate and symptom-tolerable, an effort best supported by a dietitian, with attention to fibre type and load, food texture, and cumulative dietary restriction.

### 5.5. Dietary Patterns That May Aggravate Symptoms in Functional Dyspepsia

#### 5.5.1. DASH Diet

The Dietary Approaches to Stop Hypertension (DASH) pattern shares several features with the MD, being rich in fruits, vegetables, and whole grains and low in saturated fat and added sugars but emphasises sodium restriction and was developed primarily for cardiometabolic risk reduction rather than upper gastrointestinal symptom control. No trial has evaluated the DASH pattern in patients with FD, so the evidence summarised here is indirect: it comes from controlled-feeding trials in healthy or hypertensive adults in whom bloating and fullness were secondary outcomes (interventional evidence obtained outside an FD population), and it identifies a plausible tolerability signal rather than a reason to avoid the diet. In the DASH-Sodium trial, both the DASH diet (~32 g fibre/day) and higher sodium intake were independently associated with greater odds of bloating and uncomfortable fullness [112]. The OmniHeart study, comparing a carbohydrate-rich DASH diet with protein-enriched and unsaturated fat-enriched variants, found that all three high-fibre diets increased bloating relative to baseline, with the greatest increases in the protein-rich and fat-rich arms [113]. A further controlled-feeding study similarly found that bloating increased across all DASH-style diets regardless of carbohydrate content or glycaemic index, while heartburn was more frequent with the higher-carbohydrate variants (relative risk 1.49, 95% CI 1.09 to 2.04) [115]. A GRADE-rated systematic review graded this body of evidence as moderate quality and concluded that protein-rich and unsaturated fat-rich DASH variants are likely to increase postprandial distress syndrome relative to a baseline diet [94].

These bloating signals likely reflect a higher fermentable substrate load and macronutrient-driven modulation of gastric sensorimotor and neurohormonal pathways [22,24,25]. DASH should not be dismissed when indicated for comorbid hypertension or cardiometabolic risk, but clinicians should proactively anticipate bloating in susceptible FD phenotypes and individualise fibre type, processing, and macronutrient distribution to optimise symptom tolerance without compromising cardiometabolic benefits.

#### 5.5.2. Western Dietary Patterns and Ultra-Processed Foods

Western-style dietary patterns, characterised by high intakes of saturated fat and refined carbohydrates, low fibre consumption, and frequent reliance on industrial ultra-processed foods, represent a mechanistically plausible target in FD because they combine multiple exposures that may enhance duodenal signalling and promote low-grade mucosal immune activation [116,117,118]. Within the NOVA classification framework (which classifies foods according to the extent and purpose of industrial processing), UPFs are defined as industrial formulations produced through the fractionation and recombination of food substances, typically with cosmetic additives, designed to maximise palatability and convenience [116,117,118,119,120].

Nutrient challenge data support the relevance of this dietary pattern: in controlled conditions, a high-fat meal produced significantly more nausea, epigastric pain, and postprandial fullness than an isocaloric high-carbohydrate meal in FD patients [27], consistent with the symptom profile commonly attributed to Western-style eating. A multicentre study further reported that preferences for sweet and gas-producing foods were independently associated with refractory FD and its subtypes, suggesting that Westernised eating habits may contribute to symptom persistence [121].

At a broader level, UPF-related exposures have been associated in observational studies with alterations in microbiome composition, reduced abundance of SCFA-producing taxa, impaired gut barrier integrity, and mucosal inflammation, all pathways implicated in FD pathophysiology [122]. Moreover, fast food consumption has been associated with symptom exacerbation in several studies, possibly reflecting the high fat content of these products. [123,124,125,126]. While FD-specific associations remain mixed and no interventional data exist, reducing UPF intake is clinically attractive as a safe, broadly beneficial recommendation [127].

Shifting towards a minimally processed, Mediterranean-style dietary pattern is biologically aligned with emerging FD pathophysiology, promotes nutritional adequacy, and can be personalised according to symptom tolerance without imposing unnecessary restriction [128].

## 6. Specific Foods and Beverages

The associations described in this section derive predominantly from self-reported food intolerance collected in questionnaire-based studies, and they should be read with that limitation in mind. Recall of which foods provoked symptoms is unreliable and is shaped by prevailing beliefs about what is bad for the stomach; reverse causation is difficult to exclude, because patients preferentially remember, and subsequently avoid, foods eaten during symptomatic episodes, so that a food may be reported as a trigger precisely because it happened to be eaten when symptoms occurred; and, as shown for both fat and gluten, expectancy and nocebo responses can reproduce symptoms in the absence of the suspected nutrient [59,60,61]. Blinded challenge rather than self-report is therefore required before any food can be regarded as a genuine trigger, and the frequencies quoted below should be read as descriptions of what patients believe rather than as measures of causal effect.

### 6.1. Beverages

Evidence regarding alcohol contribution to dyspeptic symptoms is conflicting. Several studies have demonstrated no significant relationship between alcohol intake and FD symptom severity across subtypes [129,130], while a large cohort study of 4390 subjects found that consumption exceeding seven alcoholic drinks per week was associated with dyspeptic symptoms [131]. Among specific alcoholic beverages, aperitifs are the most commonly implicated, with a reported food intolerance in up to 80% of dyspeptic patients, largely as aversion rather than symptom provocation, followed by wine and beer [132].

Coffee is among the most frequently cited symptom triggers, with more than 50% of patients attributing epigastric burning and heartburn to its consumption [88,132,133], and several studies reporting associations between coffee intake and dyspeptic symptoms [88,133,134], though one study found no such association [129]. Coffee increases gastric acid secretion, and the absence of a dose–response relationship in healthy controls suggests that symptom generation in FD reflects enhanced individual sensitivity rather than a direct pharmacological effect [134]. Substituting regular coffee with a non-caffeinated alternative was associated with improvement in FD symptoms, reduced reflux complaints, and normalisation of bowel habits in a small study of 51 patients [135], suggesting that non-caffeine components of coffee may also contribute. Caffeine stimulates gastric acid secretion and can transiently lower oesophageal sphincter pressure and accelerate colonic motility, which may aggravate symptoms in some patients. Many patients spontaneously reduce coffee intake in response to perceived symptom worsening, and clinicians may consider advising a structured trial of reduction or substitution in those who have not yet done so.

Milk has been reported to elicit postprandial fullness and epigastric burning in FD patients and may partly reflect its fat content [88,133]. Carbonated beverages have been reported to aggravate bloating, though the evidence supporting this association remains limited [88,124].

### 6.2. Commonly Reported Trigger Foods

Spicy foods are also frequently reported as triggers, particularly for retching and postprandial fullness [124,125,136]. The principal active compound, capsaicin, activates transient receptor potential vanilloid-1 (TRPV1) receptors involved in visceral nociception, and approximately half of FD patients exhibit chemical hypersensitivity following capsaicin challenge [137,138]. Genetic susceptibility may also contribute: the G315 polymorphism of the *TRPV1* gene has been inversely associated with FD [139]. Three cross-sectional studies reported a positive association between spicy food consumption and FD risk [68,140,141]. In one of these studies, consuming spicy foods at least three times weekly was significantly associated with postprandial fullness and nausea in a sample of 1304 participants [68]. A second study identified no significant association with FD at lower frequencies, but found a positive association with epigastric pain or burning when spicy foods were consumed more than seven times weekly [140]. The third study confirmed a positive relationship between spicy food intake and uninvestigated dyspepsia [141].

Paradoxically, capsaicin also exerts a desensitising effect on gastric nociceptive C-fibres following prolonged exposure, which may explain the finding that 2.5 g/day of red pepper powder for five weeks significantly reduced epigastric pain, fullness, and nausea in a small randomised trial of 30 FD patients, though two participants withdrew due to symptom worsening [142]_._ A single-dose capsaicin challenge (0.75 mg) has been proposed as a diagnostic test to identify gastric chemosensitivity in FD, with approximately half of patients demonstrating hypersensitivity [138]. The available clinical data are insufficient to recommend deliberate chronic spicy food ingestion as a therapeutic strategy; however, avoidance of occasional high-dose spicy food intake, which is more likely to produce acute sensitisation than desensitisation, is advisable.

### 6.3. Potentially Beneficial Foods

Evidence supporting beneficial dietary components in FD remains limited compared with other disorders of gut–brain interaction. In a small randomised controlled trial of eight FD patients, probiotic-enriched extra-virgin olive oil improved nausea and upper abdominal discomfort compared with standard olive oil, and produced greater relief of belching and postprandial fullness than antioxidant-enriched olive oil [143]. Observational data from a Middle Eastern population suggest that higher fruit intake is inversely associated with dyspeptic symptoms, with greater consumption linked to lower odds of early satiation and postprandial fullness, although some fruits are also sources of FODMAPs and may be less well tolerated by individual patients [114]. These findings are preliminary and require replication in larger, controlled studies before informing clinical recommendations.

## 7. Nutritional Concerns and Safety

**Figure 3:**
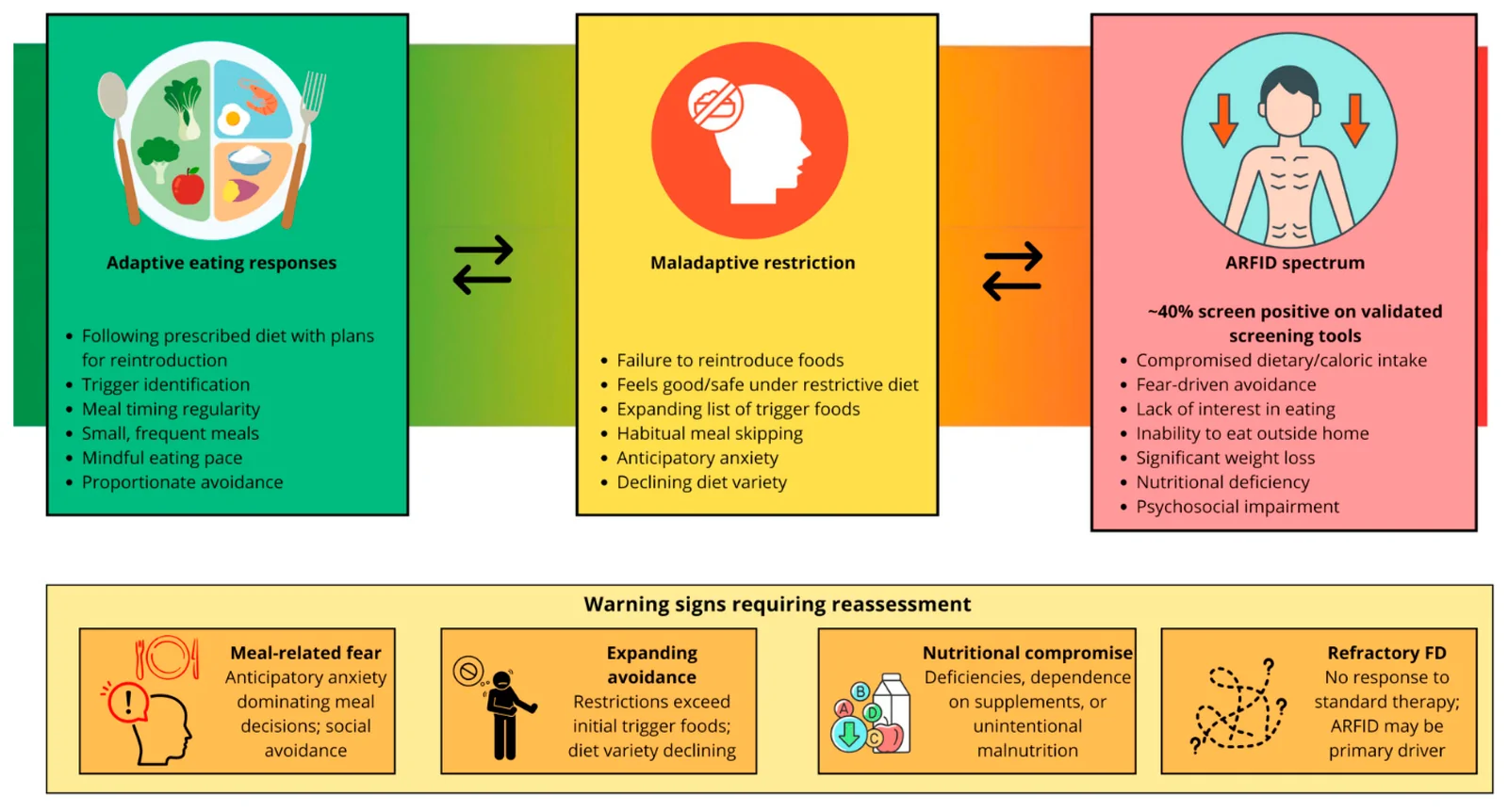
Continuum of dietary behaviour in functional dyspepsia from adaptive self-management to avoidant/restrictive food intake disorder (ARFID), with clinical warning signs. The bidirectional arrows indicate that movement along the continuum can occur in either direction. Abbreviations: ARFID: avoidant/restrictive food intake disorder; CBT: cognitive behavioural therapy; FD: functional dyspepsia.

The majority of FD patients frequently attribute their symptoms to food intake. As a result, many seek out for dietary advice or initiate self-imposed food restrictions [144]. Restrictive eating patterns may compromise nutritional adequacy, promote unintended weight loss, and in some cases contribute to maladaptive eating behaviours. The continuum of dietary behaviour in FD, ranging from adaptive self-management to avoidant/restrictive food intake disorder (ARFID), together with clinical warning signs is illustrated in Figure 3.

### 7.1. Weight Loss in Severe Dyspepsia

Although weight loss in FD might intuitively be viewed as beneficial in overweight patients, it is most often unintentional, mechanism-driven and clinically concerning rather than therapeutic, and pharmacological strategies in this subgroup are aimed at restoring rather than reducing body weight [145,146,147]. Clinically significant weight loss (>5% of body weight within 6–12 months) is common in FD, particularly in tertiary care populations, where it has been reported in approximately 35% of patients [148,149,150]. Notably, substantial weight loss (≥10 kg) has been reported in approximately 15% of patients with FD. Both impaired gastric accommodation and hypersensitivity to distention, two of the pathophysiological mechanisms described in FD, have been associated with weight loss [15,150]. Conversely, undernutrition itself can impair gastric motor function: in patients with anorexia nervosa, controlled studies have demonstrated delayed gastric emptying and prolonged intestinal transit compared with healthy controls, with normalisation in most patients upon refeeding and weight restoration [151]. Notably, these abnormalities have been documented in the context of eating disorder related malnutrition and have not been demonstrated for weight loss alone, suggesting a potentially bidirectional relationship in which severe undernutrition may perpetuate dyspeptic symptoms. Severe weight loss may prompt further diagnostic work-up [148,149,150]. Among pharmacological options, mirtazapine has shown promise in FD patients with weight loss. Studies showed improvements in dyspeptic symptoms, quality of life, and a significant weight gain [152,153,154].

### 7.2. Risk of Nutritional Deficiencies

According to the National Institute for Health and Care Excellence (NICE) guidelines, oral nutritional support should be considered in cases of unintentional weight loss exceeding 10% over the last 3–6 months, or greater than 5% if the body mass index is below 20 kg/m^2^ [155]. Conversely, the same guidelines recommend enteral tube feeding for malnourished patients with inadequate oral intake and an accessible digestive tract. Both methods of nutritional support are frequently proposed to manage involuntary weight loss in FD patients, but no studies evaluated their impact on symptoms and weight gain. In contrast, the recent position statement of the European Society of Clinical Nutrition and Metabolism (ESPEN), the European Society of Neurogastroenterology and Motility (ESNM) and the Rome Foundation, clearly advises against parenteral nutrition in patients without intestinal failure, including those with FD [156]. The risks of parenteral feeding outweigh potential benefits in this context [156].

### 7.3. Psychological and Behavioural Implications

Differentiating between FD and eating disorders can be clinically challenging. Postprandial discomfort is reported in over 90% of eating disorder patients [157,158,159]. While it remains unclear whether FD increases the risk of developing an eating disorder or severe weight loss, FD symptoms occur frequently in patients with anorexia nervosa and bulimia nervosa, and tend to improve with psychiatric treatment [160,161]. ARFID is an eating disorder previously reserved for children but is now also recognised in adults. According to the DSM-5 criteria, ARFID is characterised by restrictive eating leading to weight loss, nutritional deficiency, dependence on oral supplements or enteral feeding, and/or marked psychosocial impairment [162]. There is substantial overlap between ARFID and disorders of gut–brain interactions such as FD. Nearly 40% of patients presenting with dyspepsia or gastroparesis symptoms screen positive using validated screening tools [163]. Patients with FD may restrict food intake to prevent anticipated postprandial symptoms [164], whereas individuals with ARFID often report fear of gastrointestinal discomfort—specifically dyspepsia, nausea and vomiting—as a driver of restrictive eating behaviour. To date, the literature remains limited regarding the interplay between ARFID-like eating behaviours and restrictive dietary interventions in FD, representing an important gap that warrants further investigation.

## 8. Symptom-Based or Mechanism-Based? Deciding Whether to Start Dietary Therapy and Which One to Choose

The framework used throughout this review deserves explicit scrutiny. FD is defined by the persistence of gastroduodenal symptoms in the absence of a structural explanation, and the diagnosis is reached only once routine investigation has shown no structural, systemic or metabolic disease likely to explain the symptoms. Rome V does not prescribe a fixed work-up for this purpose: routine laboratory tests including testing for *Helicobacter pylori* are held to suffice in ordinary clinical practice, upper endoscopy is not compulsory in patients without alarm features or risk factors, and normal endoscopic findings are required only for research [1]. Symptoms alone cannot establish it, and the subdivision into PDS and EPS does not map cleanly onto any single mechanism. Impaired accommodation, delayed emptying, hypersensitivity to distension, duodenal chemosensitivity, low-grade mucosal immune activation, altered microbiota and central sensitisation are distributed unevenly among patients who meet identical criteria [14]. It is therefore legitimate to ask whether the symptom-based category is by itself sufficient. In our view, the Rome V definition remains useful as an entry criterion for trials, as a shared language between clinicians and as a way of communicating to patients that their symptoms are recognised, but it is not the endpoint of the diagnostic process.

The clearest dietary signals reviewed here appeared in clinically defined subgroups rather than in undifferentiated FD. FODMAP restriction produced greater benefit in patients with postprandial distress features, prominent bloating or FD and IBS overlap [102,104], and the first randomised comparison with traditional dietary advice in patients selected for postprandial distress syndrome showed a clear advantage for FODMAP restriction [103]. Wheat or gluten restriction produced a reproducible response in only a minority of patients with refractory symptoms, and that minority can be identified only by blinded challenge [107,108]. Symptomatic sensitivity to duodenal lipids, the best characterised mechanism in this disorder, is measurable but has not been used to select patients in any dietary trial to date [25,77]. The converse observations are equally informative: traditional dietary advice added nothing to reassurance and diagnostic explanation in a randomised trial in postprandial distress syndrome [103], and elimination guided by confocal laser endomicroscopy, the most sophisticated mechanism-directed approach tested so far, was not superior to a sham diet [43]. Stratification by symptom phenotype or by overlap therefore already carries modest but real predictive value, whereas no mechanistic marker has yet been shown in a randomised trial to predict the response to a specific diet.

**Figure 4:**
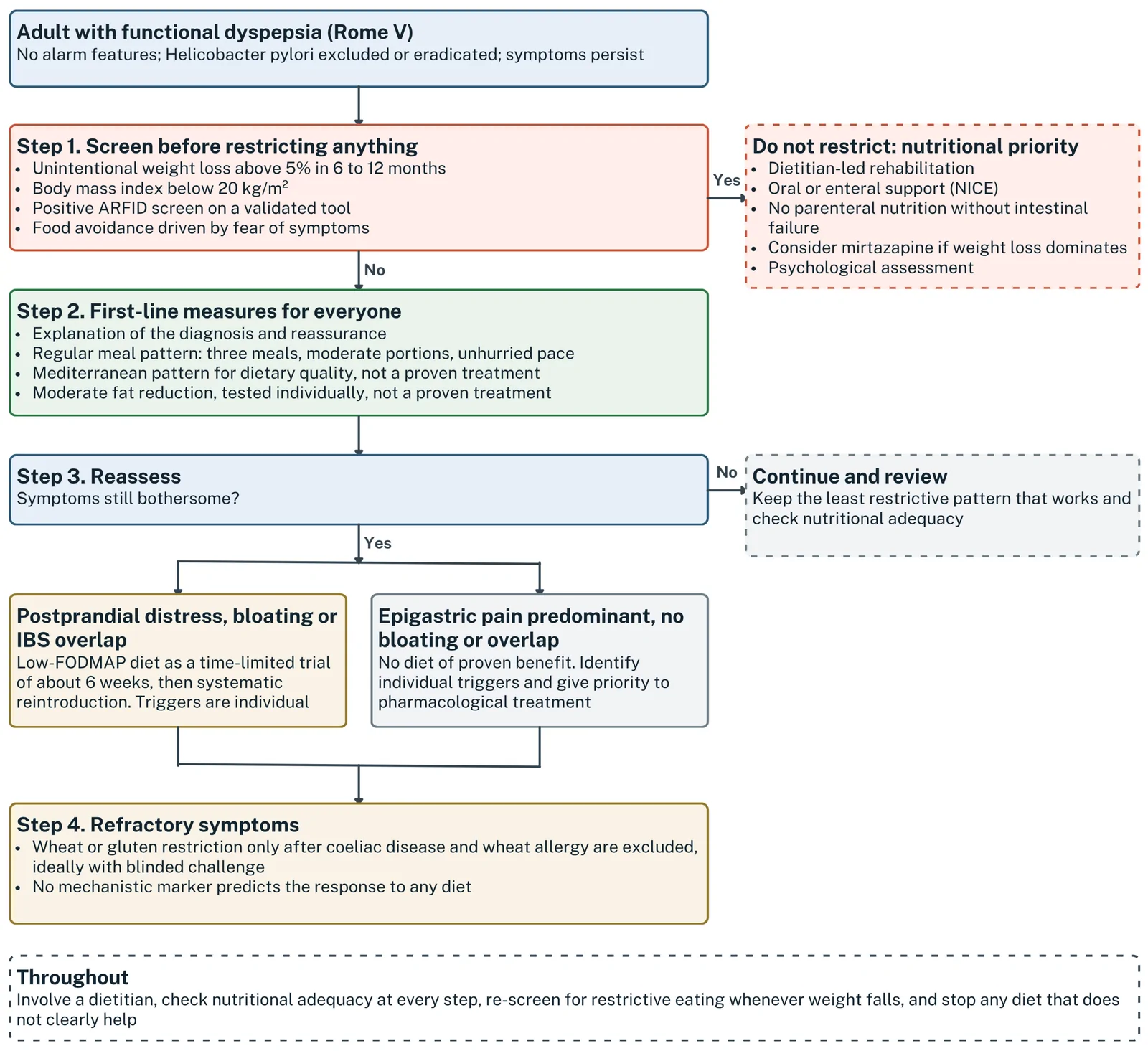
Practical sequence for deciding whether to start dietary therapy in functional dyspepsia and which one to choose. Screening for restrictive eating and nutritional risk precedes any elimination strategy; measures of dietary quality are offered to all patients; structured, time-limited trials are reserved for defined clinical phenotypes; and wheat or gluten restriction is confined to refractory patients in whom coeliac disease and wheat allergy have been excluded. None of the dietary measures shown has been validated as a treatment for FD in a long-term randomised controlled trial. Abbreviations: ARFID: avoidant/restrictive food intake disorder; FD: functional dyspepsia; FODMAP: fermentable oligosaccharides, disaccharides, monosaccharides and polyols; IBS: irritable bowel syndrome; NICE: National Institute for Health and Care Excellence.

An explicit sequence follows from this. Disordered eating and clinically significant weight loss should be excluded first, because in their presence any elimination strategy is contraindicated and the priority becomes nutritional [163,164]. An overall dietary quality framework of Mediterranean type can then be offered, since it is nutritionally adequate, safe and adjustable for meal volume, fibre type and fat load [94]. FODMAP restriction is best used as a structured, time-limited therapeutic trial followed by systematic reintroduction [99,103], and gluten or wheat restriction only in refractory patients in whom coeliac disease and wheat allergy have been excluded, ideally with blinded confirmation given the demonstrated contribution of expectancy effects [60,61,107]. The sequence is summarised in Figure 4. Future dietary trials should stratify by phenotype or by mechanism rather than randomising undifferentiated FD populations, since the discrepant results across the studies discussed here are, in our view, largely a consequence of not having done so.

## 9. Conclusions

Functional dyspepsia is a heterogeneous disorder in which diet modulates symptoms through established pathophysiological mechanisms, yet the evidence base for specific dietary interventions remains limited and subgroup-dependent. Management should therefore prioritise individualised, nutritionally adequate strategies integrated within a broader biopsychosocial framework, with early dietitian involvement and careful screening for disordered eating.

## Data Availability

No new data were created or analysed in this study. Data sharing is not applicable to this article.

## Supplementary Materials

The following supporting information can be downloaded at: https://www.mdpi.com/article/10.3390/nu18183064/s1. Table S1: Interventional studies of dietary exposures in functional dyspepsia; Table S2: Observational and mechanistic studies of dietary habits and dietary exposures in functional dyspepsia.
