# Supplementary material for: The Role of Diet in Functional Dyspepsia
Source: Nutrients. 2026 Sep 19;18(18):3064. doi: 10.3390/nu18183064 (PMC13610934; doi:10.3390/nu18183064)
Supplement: Supplementary file 1 [file nutrients-18-03064-s001.zip › nutrients-4522499-supplementary.pdf]

nutrients

MDPI

Supplementary Table S1. Interventional studies of dietary exposures in functional dyspepsia.

Study (first author, year)	Country / setting	Study design	Evidence tier	Population and sample size	Diagnostic criteria used	Dietary exposure or intervention	Comparator	Outcome measure	Main findings	Main limitations
Shiha 2026 [103]	United Kingdom, single centre	Randomised controlled trial	Interventional	60 adults with PDS (31 low-FODMAP, 29 traditional dietary advice)	Rome IV (PDS)	Low-FODMAP diet for 6 weeks after a dietetic consultation	Traditional dietary advice	PDS subscale of the Leuven Postprandial Distress Scale at thresholds of 0.5 and 0.7 points; adequate relief; quality of life	Low-FODMAP superior: response at 0.5 points 61.3% vs 37.9% (p=0.04) and at 0.7 points 61.3% vs 20.7% (p<0.001); adequate relief 71.0% vs 34.5% (p=0.005); greater improvement in quality of life and overall gastrointestinal symptoms	Single centre, 6 weeks, 78% white, participants not blinded
Buckle 2024 [70]	United Kingdom, single centre	Randomised controlled trial, open (both arms received an information leaflet)	Interventional	53 adults with PDS after a normal upper GI endoscopy (27 vs 26)	Rome IV (PDS)	Traditional dietary advice added to reassurance and diagnostic explanation for 4 weeks	Reassurance and diagnostic explanation alone	Adequate relief of dyspeptic symptoms; >0.5-point fall in the PDS subscale of the LPDS	No additional benefit: adequate relief 39% vs 33% (p=0.70); PDS subscale response 27% vs 37% (p=0.43); no difference by IBS status	Small, single centre, 4 weeks, predominantly white British, dietary changes before entry not captured
Goyal 2022 [102]	India, single centre	Randomised single-blind trial	Interventional	184 screened, 105 randomised (54 low-FODMAP, 51 traditional dietary advice)	Rome IV	Low-FODMAP diet for 4 weeks, then guided reintroduction from weeks 4 to 12	Traditional dietary advice	Short-Form Nepean Dyspepsia Index; response defined as a fall of at least 50% in the symptom score at 4 weeks	Both arms improved with no significant difference in overall response; greater improvement in the low-FODMAP arm in the subgroup with postprandial distress and bloating	Single centre, single blind, no placebo arm
Potter 2020 [86]	Australia, single tertiary centre	Pilot double-blind randomised placebo-controlled	Interventional	11 enrolled, 9 completed elimination, 4 eligible for rechallenge, 3 completed it	Rome III (FD)	Gluten-free and low-FODMAP elimination for 4 weeks, then blinded	Placebo bar	Nepean Dyspepsia Index; visual analogue symptom scores	Non-significant fall in symptoms during elimination (NDI 71.2 vs 47.1, p=0.087); no specific trigger identifiable	Severely underpowered (target 41 for the challenge phase), high attrition

Study (first author, year)	Country / setting	Study design	Evidence tier	Population and sample size	Diagnostic criteria used	Dietary exposure or intervention	Comparator	Outcome measure	Main findings	Main limitations
		dietary crossover trial				rechallenge with gluten, fructan or placebo bars				
Van de Bruaene 2026 [43]	Belgium, single tertiary centre (Leuven)	Randomised double-blind crossover sham-controlled trial	Interventional	20 included, 17 completed; 14 evaluable for the primary endpoint	Rome IV (mostly PDS)	Personalised elimination of up to two foods that produced acute mucosal changes on confocal laser endomicroscopy, 4 weeks per arm	Sham diet excluding foods that produced no acute changes	Fall of at least 0.7 points in the Leuven Postprandial Distress Scale	Acute mucosal changes in 16 of 17 patients but no advantage of the real diet over sham (4/14 vs 2/14, p=0.41); the changes did not correlate with transepithelial resistance, serum tryptase or tryptase and eosinophil-derived neurotoxin release	Recruitment stopped early (target 65), post-hoc power about 50%; food solutions applied directly to the duodenum
Shahbazkhani 2020 [107]	Iran, Tehran, two hospitals and one private clinic	Randomised double-blind placebo-controlled gluten challenge with crossover	Interventional	452 screened for dyspepsia, 193 with FD, 77 with refractory FD entered the gluten-free diet, 27 responders entered the challenge	Rome IV, refractory FD after H. pylori eradication, PPI and tricyclic or prokinetic therapy	Gluten-free diet for 6 weeks, then muffins containing about 8 g/day of gluten versus gluten-free muffins, one week each with a washout week	Gluten-free muffin	Visual analogue scales for gastrointestinal and extra-intestinal symptoms	35% (27/77) improved on the gluten-free diet; 5 relapsed on blinded challenge, that is 18.5% of responders and 6.4% of the refractory cohort; fatigue, musculoskeletal pain and headache also improved; three further patients relapsed during the 3-month follow-up despite a negative challenge	Small number entering the challenge; FODMAP intake not controlled; no histology
Goyal 2025 [102]	India, multicentre	Prospective multicentre trial with double-blind placebo-controlled gluten challenge and crossover	Interventional	252 screened, 177 entered the gluten-free diet, 154 completed it, 82 responders, 77 entered the challenge	Rome IV, refractory IBS or FD	Gluten-free diet for 6 weeks, then blinded gluten challenge	Placebo challenge	Symptom trajectory, quality of life, anxiety and depression	52.3% responded to the gluten-free diet and 20.1% worsened significantly on blinded gluten, meeting criteria for non-coeliac wheat sensitivity; female sex, FD-IBS overlap, headache, fatigue and anxiety independently predicted it; quality of life improved on the diet	Mixed IBS and FD population, so the FD-specific estimate is not separable

Study (first author, year)	Country / setting	Study design	Evidence tier	Population and sample size	Diagnostic criteria used	Dietary exposure or intervention	Comparator	Outcome measure	Main findings	Main limitations
Bortolotti 2002 [142]	Italy, Bologna	Randomised double-blind placebo-controlled parallel-group trial	Interventional	30 randomised (15 per arm); 13 and 14 completed	Clinical criteria, FD without GORD or IBS	Red pepper powder 2.5 g/day (0.7 mg capsaicin per gram) before meals for 5 weeks	Tomato powder capsules	Daily five-point Likert scores for overall and individual symptoms	Overall symptom score and the scores for epigastric pain, fullness and nausea significantly lower than placebo from the third week; about 60% fall from baseline versus under 30% with placebo; early satiety borderline (p=0.062)	Small, single centre, initial symptom worsening in 7 patients and 2 withdrawals for severe epigastric pain
Ianiro 2013 [143]	Italy, Rome	Pilot proof-of-concept blinded crossover study	Interventional	44 assessed, 10 diagnosed with FD, 8 studied	Rome III	Extra-virgin olive oil enriched with probiotics or with antioxidants added to the usual diet, 7 days per phase with 7-day washouts, then plain oil for 7 days (28 days in total)	Plain extra-virgin olive oil and antioxidant-enriched oil	Visual analogue scales for eight upper gastrointestinal symptoms	The probiotic-enriched oil improved nausea, epigastric pain or discomfort, belching, postprandial distension and postprandial fullness compared with plain oil; the antioxidant-enriched oil did not reach significance; no effect on vomiting or halitosis	Eight patients, no sample-size calculation, no placebo arm, one-week phases
Wilder-Smith 2013 [105]	Switzerland, Bern, single gastroenterology practice	Cohort with breath testing and dietary restriction	Interventional (uncontrolled)	1,372 patients with disorders of gut-brain interaction, of whom 606 with FD	Rome III; organic disease excluded by blood and stool testing (calprotectin and pancreatic elastase in all patients) and by upper and lower endoscopy with biopsies in those older than 40 years or with diarrhoea or faecal	Standardised 4-week dietary adaptation offered to patients with a positive intolerance test: 1 week low in saccharides and polyols, then weekly reintroduction of defined amounts of fructose-, fructan-, inulin- and lactose-containing foods to establish individual tolerance thresholds	None	Abdominal symptom questionnaires completed before and after the dietary change, scored on 10-point Likert scales; adequate symptom relief assessed 6 to 8 weeks after the diet was started	Adequate symptom relief in more than 80% of patients with intolerance, that is symptom induction during breath testing, and independently of malabsorption: 84% for fructose and 89% for lactose intolerance; relief was lower in patients with constipation (about 51%)	No control group, no blinding and no placebo; response self-reported; the diet was given to patients with any disorder of gut-brain interaction and the outcomes are not reported separately for the 606 patients with FD; complete outcomes in 237 of the

Study (first author, year)	Country / setting	Study design	Evidence tier	Population and sample size	Diagnostic criteria used	Dietary exposure or intervention	Comparator	Outcome measure	Main findings	Main limitations
					blood; coeliac disease excluded					312 patients who started the diet (76%)
Van den Houte 2025 [99]	Belgium, single tertiary centre (Leuven)	Open-label 6-week elimination followed by a single-blind randomised crossover reintroduction	Interventional	39 included, 36 started the diet, 27 entered the reintroduction phase	Rome IV, PDS symptoms	Strict low-FODMAP diet for 6 weeks, then 7 powders (fructans, fructose, galacto-oligosaccharides, lactose, mannitol, sorbitol) plus glucose as control, one week each	Glucose powder as control during reintroduction	Leuven Postprandial Distress Scale; duodenal transepithelial electrical resistance and dextran flux	Clinically meaningful improvement in 73% at the 0.5-point threshold and 63% at 0.7; quality of life, somatisation and depression also improved; permeability unchanged, although the change in resistance correlated with the change in symptoms (r=0.40, p=0.04); at least one FODMAP triggered recurrence in 46%, most often mannitol (23%), and 27% reacted to glucose	Single centre, open-label elimination phase, small sample, reintroduction phase not powered
Hosseinian 2024 [85]	Multinational	Systematic review and meta-analysis of trials	Interventional (synthesis)	27 studies in the review, 5 randomised trials in the meta-analysis	Mixed	Gluten challenge, gluten-free or low-gluten diet	Placebo or gluten-free diet	Severity of bloating, early satiety, epigastric pain and nausea	Gluten challenge increased bloating (weighted mean difference 0.67, 95% CI 0.37 to 0.97), early satiety (0.91, 0.58 to 1.23) and epigastric pain (0.46, 0.17 to 0.75); nausea unaffected (0.13, -0.17 to 0.43)	Substantial heterogeneity for bloating and epigastric pain; certainty of the evidence graded very low
Peng 2019 [112]	United States	Randomised controlled feeding trial (DASH-Sodium)	Interventional (non-FD population)	412 adults aged 22 years or over with untreated systolic blood pressure 120 to 159 mmHg and diastolic 80 to 95 mmHg (mean	Not applicable	DASH diet (about 32 g fibre/day) and varying sodium intake	Control diet	Bloating and uncomfortable fullness as secondary outcomes	High versus low sodium intake increased the risk of bloating regardless of diet (risk ratio 1.27, 95% CI 1.06 to 1.52) and the high-fibre DASH diet increased it across all sodium levels (1.41, 95% CI 1.22 to 1.64); the	Population without FD; bloating a secondary outcome assessed with a single question; 30-day feeding periods, so long-term

Study (first author, year)	Country / setting	Study design	Evidence tier	Population and sample size	Diagnostic criteria used	Dietary exposure or intervention	Comparator	Outcome measure	Main findings	Main limitations
				age 48 years, 57% women, 57% Black)					effect of DASH was greater in men than in women (2.04 vs 1.16, p for interaction 0.001)	effects are unknown; narrow range of sodium intake, since even the high level approximated usual US intake
Zhang 2020 [113]	United States	Randomised controlled feeding trial (OmniHeart)	Interventional (non-FD population)	164 adults aged 30 years or over with prehypertension or hypertension, free of diabetes and cardiovascular disease (mean age 53 years, 45% women, 55% Black)	Not applicable	Carbohydrate-rich, protein-enriched and unsaturated fat-enriched DASH variants	Baseline diet	Bloating	Bloating rose from 18% at baseline to 24% on the carbohydrate-rich, 33% on the protein-rich and 30% on the unsaturated fat-rich diet; only the protein-rich (relative risk 1.78, 95% CI 1.32 to 2.40) and the unsaturated fat-rich diet (1.63, 95% CI 1.17 to 2.26) significantly increased bloating over baseline, while the carbohydrate-rich diet did not (1.34, 95% CI 0.93 to 1.92); the protein-rich diet increased bloating more than the carbohydrate-rich diet (1.40, 95% CI 1.03 to 1.88), and Black participants had a higher risk on all three diets	Population without FD; bloating a secondary outcome from a single question at the end of each 6-week period; no data on coexisting irritable bowel syndrome; the soluble versus insoluble fibre and animal versus plant protein content of the diets was not measured
Wu 2021 [115]	United States	Randomised controlled feeding trial	Interventional (non-FD population)	163 adults aged 30 years or over, overweight or obese (BMI at least 25 kg/m2), with prehypertension	Not applicable	DASH-style diets differing in carbohydrate amount and glycaemic index	Usual baseline diet and the other DASH-style variants	Bloating, hunger, heartburn	Compared with the usual baseline diet, all four DASH-style diets increased bloating (relative risk 1.59, 95% CI 1.25 to 2.00) and loose stools, and reduced heartburn (0.60, 95% CI	Population without FD; gastrointestinal symptoms were secondary outcomes rated only for occurrence and

https://doi.org/10.3390/nu18183064

**Study (first author, year)**	**Country / setting**	**Study design**	**Evidence tier**	**Population and sample size**	**Diagnostic criteria used**	**Dietary exposure or intervention**	**Comparator**	**Outcome measure**	**Main findings**	**Main limitations**
				or hypertension; 5 weeks per diet with a 2-week washout					0.43 to 0.82); between diets, the higher-carbohydrate variants increased heartburn (1.49, 95% CI 1.09 to 2.04), hunger (1.16, 95% CI 1.04 to 1.30) and diet satisfaction (1.10, 95% CI 1.01 to 1.20), with no between-diet difference in bloating	severity, without frequency; 5-week feeding periods; hunger and diet satisfaction each rated with a single 9-point question; open feeding trial at high risk of bias for lack of blinding

https://doi.org/10.3390/nu18183064

Supplementary Table S2. Observational and mechanistic studies of dietary habits and dietary exposures in functional dyspepsia.

Study (first author, year)	Country / setting	Study design	Evidence tier	Population and sample size	Diagnostic criteria used	Dietary exposure or intervention	Comparator	Outcome measure	Main findings	Main limitations
Ostadsharif 2026 [66]	Multinational, predominantly Middle East and East Asia	Systematic review and meta-analysis of observational studies	Observational (synthesis)	11 studies, 21,220 participants	Mixed Rome versions	Dietary habits: meal frequency, regularity, meal skipping, eating speed	Not applicable	Risk of FD	Spicy food intake was associated with a higher risk of FD (4 studies, OR 1.32, 95% CI 1.04 to 1.67), of epigastric pain (4 studies, OR 1.46, 95% CI 1.22 to 1.75) and of epigastric burning (4 studies, OR 1.47, 95% CI 1.21 to 1.78), but not with early satiety or postprandial fullness; higher meal frequency was associated with a lower risk of FD (2 studies, OR 0.52, 95% CI 0.32 to 0.86)	All included studies cross-sectional; heterogeneous exposure definitions; only two to four studies contribute to each pooled estimate
Hassanzadeh 2016 [67]	Iran (SEPAHAN)	Cross-sectional, population based	Observational	More than 4,700 adults	Modified Rome III, uninvestigated dyspepsia	Number of daily meals, meal skipping, eating speed, fluid intake with meals	Not applicable	Prevalence of uninvestigated dyspepsia	Inverse association between number of daily meals and FD prevalence, lowest with three or more regular meals per day; eating a main meal in under 10 minutes associated with 42% higher odds; skipping breakfast or lunch positively associated; intra-meal fluid intake associated only in overweight participants	Cross-sectional, self-reported diet, no endoscopy
Xu 2017 [68]	China, rural South China	Cross-sectional	Observational	1,304 participants	Rome III, using a Chinese-language questionnaire modified	Irregular mealtimes, meal	Not applicable	FD and its subtypes	Irregular mealtimes associated with both PDS and EPS; spicy food at least	Cross-sectional, single rural area

https://doi.org/10.3390/nu18183064

Study (first author, year)	Country / setting	Study design	Evidence tier	Population and sample size	Diagnostic criteria used	Dietary exposure or intervention	Comparator	Outcome measure	Main findings	Main limitations
					from the Rome III criteria; gastroscopy, blood and stool tests, ultrasound and H. pylori testing performed to exclude organic disease	skipping, spicy food, late-night eating			three times weekly associated with postprandial fullness, nausea and vomiting	
Yamamoto 2022 [69]	Japan, university students	Cross-sectional	Observational	8,913 students (the abstract of the paper states 8,923)	Rome III	Meal frequency, skipping breakfast, lunch or dinner, snacks and midnight snacks	Not applicable	Prevalence of FD	Skipping breakfast (adjusted OR 1.60, 95% CI 1.10 to 2.32) and lunch (2.52, 95% CI 1.04 to 5.18) positively associated with FD; meal frequency inversely associated (adjusted OR 2.72 for one meal per day and 1.69 for two, versus three; p for trend 0.001); no association with snacks	Cross-sectional; young students only; no data on energy intake or eating speed
Agah 2022 [81]	Iran (SEPAHAN)	Cross-sectional, population based	Observational	4,763 adults	Modified Rome III, uninvestigated dyspepsia	Macronutrient intake by food-frequency questionnaire	Not applicable	Uninvestigated dyspepsia and its individual symptoms	Epigastric pain more prevalent in the highest protein category (>20% of energy) than the lowest (50% vs 39.7%, p=0.02), not retained after adjustment (OR 1.48, 95% CI 0.84 to 2.58); carbohydrate 48.2% vs 49.2% of energy (p=0.01), adjusted OR 0.86 (0.64 to 1.16) for intake above 55% of energy	Cross-sectional, food-frequency questionnaire, no endoscopy, single professional cohort
Pilichiewicz 2009 [82]	Australia	Prospective observational study	Observational	20 patients with FD and 21 healthy subjects	Rome II (Rome III not yet available when the study began); organic causes excluded by	Diet diary	Healthy subjects (for the comparison of	Postprandial fullness	Postprandial fullness related directly to fat and protein intake and inversely to carbohydrate intake	Very small sample

Study (first author, year)	Country / setting	Study design	Evidence tier	Population and sample size	Diagnostic criteria used	Dietary exposure or intervention	Comparator	Outcome measure	Main findings	Main limitations
					upper endoscopy and abdominal ultrasound in the previous 12 months, H. pylori negative		intake and eating patterns)			
Staudacher 2021 [104]	Australia, Brisbane, single tertiary outpatient dietetic clinic	Retrospective observational service evaluation of clinical notes	Observational	59 consecutive adults with FD (40 low-FODMAP advice, 19 standard advice); 48 of 59 (81%) had coexisting irritable bowel syndrome	No formal Rome criteria applied: FD stated on the dietetic referral or documented in the medical history, or at least three of the seven epigastric items of the SAGIS scored moderate or higher	Low-FODMAP dietary advice	Standard dietetic advice	Structured Assessment of Gastrointestinal Symptom Scale, epigastric domain (maximum 28) and total score (maximum 88); response defined as a fall of at least 30%	Greater fall in the epigastric domain with low-FODMAP advice (estimated marginal mean -3.6, 95% CI -4.9 to -2.2, vs -0.9, 95% CI -2.9 to 1.1; p=0.032) and in the total score (-9.4 vs -3.3; p=0.026); more responders both on the epigastric domain (50% vs 16%, p=0.012) and on the total score (55% vs 26%, p=0.039); adherence similar in the two groups	Not randomised; retrospective data extracted from clinical notes in a single tertiary centre; 81% had coexisting irritable bowel syndrome, so the effect on FD symptoms alone cannot be isolated; FD subtype and duration of follow-up not recorded; possible selection bias if non-responders did not return
Van de Bruaene 2025 [5]	Belgium, Leuven (coordinating centre), with a local open-access endoscopy cohort, a national outpatient cohort, the	Pooled analysis of four FD datasets	Observational	1,337 patients with FD from four independent databases: 971 across three questionnaire cohorts (223 open-access endoscopy, 410 outpatient clinic, 483 of 1,074 AEGIS	Rome IV: EPS defined as epigastric pain or burning at least once a week for at least 6 months, PDS as postprandial fullness or early satiation on at least 3 days a week for 6 months; pain counted as meal-related when it followed the	Not applicable (symptom phenotyping)	Not applicable	Categorisation into postprandial EPS, meal-unrelated EPS and PDS with or without postprandial epigastric pain, with associated	Under Rome IV the distribution was 896 PDS, 252 EPS and 186 overlap; postprandial epigastric pain without PDS symptoms was present in about 40% of patients with EPS (32% to 38% in the three questionnaire cohorts and 59% of those with isolated epigastric pain in the breath test	Pooled analysis of existing datasets from secondary and tertiary care, so symptoms are likely more severe than in the community; questionnaire design differed between datasets; the breath test cohort was

Study (first author, year)	Country / setting	Study design	Evidence tier	Population and sample size	Diagnostic criteria used	Dietary exposure or intervention	Comparator	Outcome measure	Main findings	Main limitations
	international AEGIS network (16 European and Asian centres) and a Leuven gastric emptying breath test cohort			participants meeting FD criteria) and 498 referred for a gastric emptying breath test	meal often or in more than 50% of episodes, or, in the breath test cohort, when it appeared or rose by at least one point within 120 minutes of the meal			symptoms and gastric emptying compared between categories	cohort), and half of the patients with PDS reported postprandial epigastric pain; the latter group had the highest burden of bloating, heartburn, nausea and dysphagia	probably enriched in postprandial symptom reporting; splitting large datasets into smaller categories reduces power; the pathophysiology and treatment response of the new category have not been studied
Ashoorion 2025 [94]	Multinational	GRADE-rated systematic review of dietary patterns	Observational and interventional (synthesis)	7 studies (3 randomised trials, 4 observational)	Mixed	Mediterranean, DASH, vegetarian and vegan patterns	Usual or baseline diet	FD and postprandial distress syndrome	High versus low Mediterranean diet adherence associated with a lower likelihood of FD (OR 0.41, 95% CI 0.25 to 0.68; 151 fewer cases per 1000, 95% CI 74 to 203 fewer), from a single low-risk-of-bias observational survey of 1,134 participants, moderate certainty; protein-rich and unsaturated fat-rich DASH variants likely to increase PDS; effect of vegetarian and vegan diets uncertain	Few studies; the Mediterranean estimate rests on one observational study
Cooke 2023 [100]	Online survey, mostly Australia, United Kingdom and North America	Cross-sectional survey with healthy controls	Observational	121 adults with dyspepsia and 52 healthy controls	Rome IV, self-reported, no endoscopic confirmation	Dietary habits, perceived triggers and restrictions, nutrient intake by food-	Healthy controls	Perceived triggers, special diets, nutrient intake, quality of	FODMAPs were the most reported trigger (55%); 88% of the dyspepsia group had followed a special diet versus 47% of controls, most often low-FODMAP (69%); the dyspepsia group	Convenience online sampling, self-reported diagnosis, marked sex imbalance, recruitment partly through

https://doi.org/10.3390/nu18183064

Study (first author, year)	Country / setting	Study design	Evidence tier	Population and sample size	Diagnostic criteria used	Dietary exposure or intervention	Comparator	Outcome measure	Main findings	Main limitations
						frequency questionnaire		life, salivary cortisol	consumed less fibre (p=0.014), calcium (p=0.015) and total FODMAPs (p<0.001); no difference in meal or snack frequency or eating speed	a FODMAP-related channel
Tabibian 2021 [114]	Iran, Isfahan (SEPAHAN)	Cross-sectional	Observational	3,362 participants	Modified Persian version of the Rome III questionnaire; intake assessed with a validated 106-item dish-based food-frequency questionnaire	Fruit and vegetable intake	Not applicable	FD and its symptoms	Prevalence of FD 14.5%; the highest tertile of fruit intake was associated with 32% lower odds of FD (OR 0.68, 95% CI 0.51 to 0.90) and with lower odds of early satiation (0.67, 95% CI 0.54 to 0.85) and postprandial fullness (0.75, 95% CI 0.61 to 0.92); vegetable intake was not associated with FD overall, but was in men (0.53, 95% CI 0.31 to 0.91)	Cross-sectional, so reverse causation is possible, since patients may eat less fruit and vegetables because of their symptoms; self-administered food-frequency questionnaire; non-academic staff of a single university, so generalisability is limited; residual confounding
Jiang 2014 [121]	China, Guangdong Province, four hospitals	Prospective observational study	Observational	1,341 outpatients with FD who completed the questionnaire (327 refractory, 1,014 non-refractory) and 100 healthy volunteers	Rome III, with endoscopy, laboratory tests and abdominal imaging to exclude organic disease; refractory FD defined as continuous symptoms for at least 6 months unresponsive to at least two classes of drug	Unhealthy dietary behaviour, preference for sweet and gas-producing foods	Not applicable	Refractory FD and its subtypes	On logistic regression, skipping meals (OR 1.64, 95% CI 1.18 to 2.27), eating extra meals (1.63, 95% CI 1.02 to 2.60) and a preference for sweet (1.35, 95% CI 1.04 to 1.76) and gas-producing food (1.54, 95% CI 1.02 to 2.31) were associated with refractory FD; within subtypes, spicy food was preferred in refractory EPS and sweet	Retrospective review of medical records using a self-designed, unvalidated questionnaire; outpatients of four hospitals in a single province; dietary behaviour recorded only for the month before diagnosis; cross-sectional

Study (first author, year)	Country / setting	Study design	Evidence tier	Population and sample size	Diagnostic criteria used	Dietary exposure or intervention	Comparator	Outcome measure	Main findings	Main limitations
									and gas-producing food in refractory PDS	comparison, so causality cannot be inferred
Colak 2022 [123]	Turkey, single endoscopy unit	Descriptive cross-sectional	Observational	60 patients with FD (71.7% women, mean age 43.8 years)	Rome IV	Eating habits, cooking methods, smoking, alcohol and a 51-item food-frequency questionnaire	Not applicable (within-cohort comparison by symptom frequency)	Frequency of postprandial fullness, early satiety, epigastric pain and burning	Postprandial fullness more frequent in those always using roasting (p=0.013); lower intake of broccoli, radish, celery, green olives and olive oil in those with more fullness; lower intake of dried fruit, green olives, butter, fast food and alcohol in those with more epigastric pain; no association with meal frequency, eating duration or chewing speed	Small single-centre sample, one food record, avoidance behaviour makes the direction of the associations uncertain
Akhondi-Meybodi 2015 [124]	Iran, Yazd, hospital gastroenterology department	Cross-sectional questionnaire study	Observational	384 patients (60.4% women, mean age 39.2 years)	Rome III, endoscopy performed in all	Self-reported aggravating or relieving effect of 114 individual foods	Not applicable	Patient-reported aggravation or relief of dyspeptic symptoms	Most aggravating: sausage and bologna, pickles and vinegar, soft drinks, grains, tea, salty foods, pizza, watermelon, red pepper and pasta; most relieving: apple, rice, rock candy, bread, caraway, dates, honey, yogurt, quince and walnut	Entirely self-reported and shaped by local food culture; cross-sectional; patients with GORD not excluded
Göktaş 2016 [125]	Turkey, five outpatient gastroenterology clinics	Comparative cross-sectional study with healthy controls	Observational	168 adults with FD (70 EPS, 61 PDS, 37 mixed) and 135 healthy controls	Rome III	Questionnaire and short-form food-frequency questionnaire on meal and snack	Healthy controls	Self-reported symptom-triggering foods and meal patterns	Most frequent triggers were fried and fatty foods (27.1%), hot spices (26.4%) and carbonated drinks (21.8%); carbonated drinks (37.3% vs 25.7% and 22.1%, p=0.006) and	Self-reported triggers, cross-sectional, culture-specific food list

Study (first author, year)	Country / setting	Study design	Evidence tier	Population and sample size	Diagnostic criteria used	Dietary exposure or intervention	Comparator	Outcome measure	Main findings	Main limitations
						frequency, fluid intake and trigger foods			legumes (18.6%, p=0.02) triggered symptoms more often in PDS; no difference in main-meal or snack frequency between any group; BMI slightly higher in FD (26.1 vs 24.6 kg/m2, p=0.005)	
Chirila 2016 [126]	Romania, Iasi, general practice, urban population sample	Cross-sectional population-based interview study	Observational	184 adults (participation 73.6%), 14 with FD and 57 with GORD	Rome III for FD, Montreal for GORD	Eating habits and food-frequency questionnaire based on the EPIC protocol	Participants without the disorder	Prevalence of FD and GORD and their predictors	FD in 7.6% and GORD in 31.0%, overlapping in 22.4%; on multivariable analysis the predictors of FD were low educational level (OR 22.4, 95% CI 3.4 to 150.1), consumption of canned food (OR 2.38) and alcohol at least weekly (OR 5.4, 95% CI 1.2 to 23.6); no eating habit was associated with either disorder	Only 14 patients with FD, so the estimates are very imprecise; cross-sectional; type of canned food not recorded
Saneei 2016 [140]	Iran, Isfahan Province (SEPAHAN)	Cross-sectional	Observational	4,763 adults	Modified Persian version of the Rome III questionnaire; chronic uninvestigated dyspepsia, without clinical or endoscopic assessment	Spicy food intake	Not applicable	Chronic uninvestigated dyspepsia	Prevalence of chronic uninvestigated dyspepsia 15%; intake of spicy food 10 or more times a week was associated with dyspepsia (OR 1.64, 95% CI 1.09 to 2.49 crude; 1.68, 95% CI 1.01 to 2.79 after adjustment for diet-related practices) and with epigastric pain or burning (adjusted OR 1.62, 95% CI 1.12 to 2.35, and 1.54, 95% CI 1.09 to 2.18 at 7 to 9 times weekly);	Cross-sectional; a single self-reported question on the frequency of mixed spicy foods, with the quantity consumed not recorded; no clinical or endoscopic assessment; non-academic staff of a medical university

https://doi.org/10.3390/nu18183064

Study (first author, year)	Country / setting	Study design	Evidence tier	Population and sample size	Diagnostic criteria used	Dietary exposure or intervention	Comparator	Outcome measure	Main findings	Main limitations
									the association with postprandial fullness did not persist after adjustment and there was none with early satiation	
Li F 2014 [141]	China, Chinese navy personnel in active service	Cross-sectional questionnaire survey	Observational	8,600 surveyed, 8,106 responded (94.3%), 4,899 analysed (1,046 offshore, 3,853 onshore), 397 with uninvestigated dyspepsia	Chinese version of the Rome III questionnaire, uninvestigated dyspepsia (no endoscopy)	Eating habits including preference for spicy, cold, hot and hard food, overeating and picky eating; lifestyle and offshore training	Onshore personnel (offshore training under 1 month per year)	Prevalence of uninvestigated dyspepsia and of its subtypes	Overall prevalence 8.1%, higher offshore than onshore (12.6% vs 6.9%, p<0.001; OR 1.955 for dyspepsia and 1.789 for PDS); on univariate analysis preference for spicy food, overeating, picky eating and preference for cold, hot or hard food were all associated with dyspepsia, and on multivariate analysis preference for spicy food remained independently associated (OR 2.236, p=0.018) together with family history and short sleep; the final model retained offshore training (OR 1.580), family history (OR 1.765) and smoking (OR 1.270), but not alcohol	Cross-sectional; no endoscopy or blood tests; young, mostly male and single military population, so not generalisable; offshore duration recorded only in three brackets
Carvalho 2010 [88]	Brazil	Case-control	Observational	41 patients with FD and 30 healthy volunteers	Clinical criteria, Rome criteria not applied: dyspeptic symptoms for at least 6 months in the preceding year with normal upper endoscopy, abdominal ultrasound and blood glucose;	Food intolerance, diet composition, eating patterns	Healthy controls	Reported food intolerance and macronutrient intake	No significant difference in total energy or in absolute carbohydrate intake between patients and controls, although patients took a higher proportion of energy as carbohydrate (median 56% vs 50%, p=0.001) and a	Small single-centre sample (41 patients and 30 healthy volunteers); 7-day food diary and self-reported intolerance; avoidance behaviour makes the

Study (first author, year)	Country / setting	Study design	Evidence tier	Population and sample size	Diagnostic criteria used	Dietary exposure or intervention	Comparator	Outcome measure	Main findings	Main limitations
					irritable bowel syndrome excluded				lower proportion as fat (28% vs 34%, p=0.001); the foods most often linked to symptoms were carbonated drinks (63.4%), fried foods (58.5%), bell pepper (56.1%), sausage and coffee (53.6% each)	direction of the associations uncertain
Mullan 1994 [87]	Ireland	Case-control	Observational	40 patients with functional dyspepsia, 40 with organic dyspepsia and 80 healthy controls (160 in total)	No Rome criteria; entry required a dyspeptic symptom score above 4 on a 0 to 3 frequency and severity scale, with endoscopy separating functional from organic dyspepsia	Food and nutrient intake, eating patterns	Patients with organic dyspepsia and healthy controls	Nutrient intake	As reported by later reviews, since the original could not be retrieved: intake of energy, carbohydrate, fat and protein was lower in women with functional dyspepsia than in healthy controls, with no difference in men, so the lower carbohydrate intake is probably confounded by the lower total energy intake	Old study, small, no Rome criteria; findings restricted to women; likely confounding by total energy intake; 7-day food and symptom diary
Yamamoto 2022 [69]	Japan, university students	Cross-sectional	Observational	8,923 university students, of whom 168 (1.9%) had FD	Rome III	Sleep duration and sleep disturbance (not a dietary exposure)	Not applicable	Prevalence of FD and its subtypes	All types of sleep disturbance were independently associated with FD (total sleep disturbance adjusted OR 4.11, 95% CI 2.89 to 5.78), whereas self-reported sleep duration was not; include only if the tables are meant to cover lifestyle as well as diet	Cross-sectional; no endoscopy data available, so unrecognised organic disease cannot be excluded; no data on mental illness; sleep duration and disturbance not assessed with a validated questionnaire; young students only

https://doi.org/10.3390/nu18183064

Study (first author, year)	Country / setting	Study design	Evidence tier	Population and sample size	Diagnostic criteria used	Dietary exposure or intervention	Comparator	Outcome measure	Main findings	Main limitations
Tack 2003 [74]	Belgium, Leuven, tertiary referral motility clinic	Physiological study, satiety drinking test	Mechanistic	37 patients with severe FD and 25 healthy controls in the main comparison (27 healthy volunteers took part across the study protocols)	Dyspeptic symptoms for at least 3 months in the absence of organic, systemic or metabolic disease (Rome I duration criteria), present on at least 3 days a week with two or more symptoms rated relevant or severe; normal endoscopy, biochemistry and upper abdominal ultrasound; oesophagitis, erosive lesions, predominant heartburn and previous peptic ulcer excluded	Nutrient drinking test	Healthy controls	Amount of liquid meal (kcal and volume) ingested at maximum satiety during a slow caloric drinking test, with gastric barostat measurement of accommodation and sensitivity and a gastric emptying breath test	Maximum satiety occurred at a much lower intake in patients than in controls (542 ± 50 vs 1508 ± 53 kcal, that is 361 vs 1005 ml, p<0.0001); the endpoint correlated with meal-induced accommodation (r=0.71 in patients, p<0.001) but not, on multivariate analysis, with gastric emptying or sensitivity to distension, and predicted impaired accommodation with 92% sensitivity and 86% specificity	Small single-centre study in tertiary-referred patients with severe, meal-related symptoms, so not generalisable to unselected dyspepsia; six patients had previously required nutritional support; H. pylori status of the controls not available
